# Transcriptomic and proteomic changes associated with cobalamin-dependent propionate production by the rumen bacterium *Xylanibacter ruminicola*

**DOI:** 10.1128/msystems.00864-24

**Published:** 2024-10-29

**Authors:** Sam C. Mahoney-Kurpe, Nikola Palevich, Dragana Gagic, Patrick J. Biggs, Peter M. Reid, Ianina Altshuler, Phillip B. Pope, Graeme T. Attwood, Christina D. Moon

**Affiliations:** 1 AgResearch Ltd, Grasslands Research Centre, Palmerston North, New Zealand; 2 School of Food Technology and Natural Sciences, Massey University, Palmerston North, New Zealand; 3 School of Veterinary Science, Massey University, Palmerston North, New Zealand; 4 Faculty of Biosciences, Norwegian University of Life Sciences, Ǎs, Norway; 5 MACE Laboratory, Alpine and Polar Environmental Research Centre (ALPOLE), École Polytechnique Fédérale de Lausanne (EPFL), Lausanne, Switzerland; 6 Faculty of Chemistry, Biotechnology and Food Science, Norwegian University of Life Sciences, Ǎs, Norway; 7 Centre for Microbiome Research, School of Biomedical Sciences, Queensland University of Technology (QUT), Translational Research Institute, Woolloongabba, Queensland, Australia; The University of Maine, Orono, Maine, USA

**Keywords:** fermentation, short-chain fatty acids, vitamin B_12_

## Abstract

*Xylanibacter ruminicola* is an abundant rumen bacterium that produces propionate in a cobalamin (vitamin B_12_)-dependent manner via the succinate pathway. However, the extent to which this occurs across ruminal *Xylanibacter* and closely related bacteria, and the effect of cobalamin supplementation on the expression of propionate pathway genes and enzymes has yet to be investigated. To assess this, we screened 14 strains and found that almost all strains produced propionate when supplemented with cobalamin. *X. ruminicola* KHP1 was selected for further study, including complete genome sequencing, and comparative transcriptomics and proteomics of KHP1 cultures grown with and without supplemented cobalamin. The complete KHP1 genome was searched for cobalamin-binding riboswitches and four were predicted, though none were closely located to any of the succinate pathway genes, which were dispersed at numerous genomic loci. Cobalamin supplementation led to the differential expression of 17.5% of genes, including genes encoding the cobalamin-dependent methylmalonyl-CoA mutase and some methylmalonyl-CoA decarboxylase subunits, but most propionate biosynthesis pathway genes were not differentially expressed. The effect of cobalamin supplementation on the KHP1 proteome was much less pronounced, with the only differentially abundant propionate pathway enzyme being methylmalonyl-CoA mutase, which had greater abundance when supplemented with cobalamin. Our results demonstrate that cobalamin supplementation does not result in induction of the entire propionate biosynthesis pathway, but consistently increased expression of methylmalonyl-CoA mutase at transcriptome and proteome levels. The magnitude of the differential expression of propionate pathway genes observed was minor compared to that of genes proximate to predicted cobalamin riboswitches.

**IMPORTANCE:**

In ruminants, the rumen microbial community plays a critical role in nutrition through the fermentation of feed to provide vital energy substrates for the host animal. Propionate is a major rumen fermentation end-product and increasing its production is desirable given its importance in host glucose production and impact on greenhouse gas production. Vitamin B_12_ (cobalamin) can induce propionate production in the prominent rumen bacterium *Xylanibacter ruminicola*, but it is not fully understood how cobalamin regulates propionate pathway activity. Contrary to expectation, we found that cobalamin supplementation had little effect on propionate pathway expression at transcriptome and proteome levels, with minor upregulation of genes encoding the cobalamin-dependent enzyme of the pathway. These findings provide new insights into factors that regulate propionate production and suggest that cobalamin-dependent propionate production by *X. ruminicola* is controlled post-translationally.

## INTRODUCTION

Ruminant animals are characterized by the presence of a fermentative forestomach, the rumen, which hosts a complex community of microbes. The rumen microbiota drives ruminant nutrition and is primarily responsible for the degradation of dietary plant polysaccharides, and their fermentation into short-chain fatty acids (SCFAs) that the host uses to satisfy most of its energy requirements (1). Of the three major SCFAs (acetate, propionate, and butyrate), propionate is of particular importance to animals with high energy requirements as it is the major precursor of host gluconeogenesis (2). Propionate also acts as an alternative hydrogen sink to methane production in rumen metabolism (3). Enhancement of ruminal propionate production is therefore desired, particularly in forage-fed animals where relative propionate levels are generally lower than in animals fed concentrate-based diets which are more readily digestible (4, 5). Propionate formation from sugars mainly proceeds by either the acrylate pathway, in which lactate is an intermediate, or the randomizing succinate pathway (6). Propionate is also produced to a lesser extent via the propanediol pathway from the deoxy sugars fucose and rhamnose, or alternatively from dihydroxyacetone phosphate or lactate (7).

Ruminal *Xylanibacter* (8) were formerly classified as members of the genus *Prevotella* (9), an important and abundant rumen bacterial group in ruminants globally (10). *Xylanibacter ruminicola*, first described as *Bacteroides ruminicola* in 1958 with type strain 23^T^ (11)*,* is among the most extensively characterized species of rumen bacteria. Upon isolation, strain 23^T^ was observed to produce succinate but not propionate (11). Later work showed propionate production of *X. ruminicola* cultures grown in medium containing 40% (vol/vol) rumen fluid, but its production markedly reduced in media containing only 20% (vol/vol) rumen fluid (12). Propionate production by *X. ruminicola* 23^T^ was shown to be dependent on cobalamin (vitamin B_12_) supplementation, with succinate accumulating in its absence (13). Given the requirement of the succinate pathway enzyme methylmalonyl-CoA mutase for cobalamin as a cofactor (14), these observations are consistent with propionate production in *Xylanibacter* via the succinate pathway. The presence of succinate pathway genes has subsequently been confirmed in *X. ruminicola* by genomic (15) and proteomic analyses (16).

The specific genes and enzymes involved in the succinate pathway of propionate production in *X. ruminicola* are now quite well-defined (16). Phosphoenolpyruvate is converted to succinate via oxaloacetate, malate, and fumarate in a series of steps catalyzed by phosphoenolpyruvate carboxykinase (EC 4.1.1.49), malate dehydrogenase (EC 1.1.1.37), fumarate hydratase (EC 4.2.1.2), and fumarate reductase (EC 1.3.5.1 and 1.3.5.4) enzymes. Succinate is then converted to succinyl-CoA via a succinate:propionate CoA-transferase (EC 2.8.3.27), which is coupled to the final step of the pathway converting propionyl-CoA to propionate. Succinyl-CoA is converted to *R* and *S*-methylmalonyl-CoA via methylmalonyl-CoA mutase (EC 5.4.99.2) and methylmalonyl-CoA epimerase (EC 5.1.99.1), respectively, while the conversion of *S*-methylmalonyl-CoA to propionyl-CoA occurs via a sodium ion translocating methylmalonyl-CoA decarboxylase (EC 7.2.4.3) (17), rather than by transcarboxylase as occurs in propionibacteria. However, *X. ruminicola* appears to lack a gene encoding a delta subunit of methylmalonyl-CoA decarboxylase (MmcD) shown to be indispensable to formation of the complex in the characterized methylmalonyl-CoA decarboxylase of *Veillonella parvula* (18). It was recently also shown that in ruminal *Xylanibacter*, Rnf, a ferredoxin-NAD+ oxidoreductase (EC 7.2.1.2) (19), is also required to balance redox cofactors generated during fermentation via the succinate pathway (16).

Mechanisms potentially regulating the cobalamin-dependent propionate pathway expression of *X. ruminicola* are not well understood. As methylmalonyl-CoA mutase requires cobalamin as a cofactor, it is possible that the regulation of cobalamin-induced propionate production occurs post-translationally simply by the presence or absence of cobalamin rendering the expressed methylmalonyl-CoA mutase as active or inactive, respectively. However, cobalamin has also been shown to regulate gene expression via cobalamin-binding riboswitches, which are elements widespread across bacteria (20), typically in 5′ UTR regions of mRNAs that bind cobalamin to regulate expression of nearby genes (21). Most cobalamin riboswitches identified to date function by the downregulation of genes upon cobalamin binding, such as those involved in cobalamin uptake (22) or biosynthesis (23) as a means of energy conservation when intracellular cobalamin concentrations are sufficient. However, nonclassical mechanisms of cobalamin riboswitch function have also been identified, such as a cobalamin riboswitch identified in *Listeria monocytogenes* that controls expression of an antisense RNA, resulting in genes involved in propanediol catabolism being maximally expressed only in the presence of cobalamin (24). However, whether any similar mechanism might regulate cobalamin-dependent propionate production in ruminal *Xylanibacter* has not been investigated.

The aim of this study was to assess the extent to which cobalamin induction of propionate production occurs among ruminal *Xylanibacter* strains. Moreover, we wanted to investigate the impact of cobalamin on the expression of genes involved in propionate production and gain insight into the regulatory mechanisms involved. The ability of *X. ruminicola* to be cultured in both the presence and absence of cobalamin supplementation (13) provided a model for investigation. We therefore undertook comparative transcriptomic and proteomic analyses of *X. ruminicola* grown with and without supplemented cobalamin, to gain greater insight into the impact of cobalamin on propionate pathway expression in *X. ruminicola*.

## MATERIALS AND METHODS

### Biological material and growth conditions

All rumen bacterial strains included in this study were sourced from the Hungate1000 collection (15) and cultivated under standard anaerobic methods (25). Cultures were revived from frozen glycerol stocks in anaerobically prepared nutrient-rich M2GSC medium (26) containing 30% (vol/vol) centrifuged rumen fluid, and 0.2% (wt/vol) each of glucose, cellobiose, and soluble starch. Cultures were passaged three times on the medium described by Strobel (13) containing 0.2% (wt/vol) glucose in the absence of supplemented cobalamin, to minimize potential carry-over effects of cobalamin present in rumen fluid of the revival medium. Cultures were incubated as static batch liquid cultures at 39°C in the dark.

### Screening of “*Prevotella* 1” strains for cobalamin-dependent propionate production

Selected strains of the Hungate1000 collection that were assigned to the “*Prevotella* 1” genus-level cluster originally described by Henderson et al. (27), were revived from frozen glycerol stocks, and their identities and purity were confirmed by Gram staining and 16S rRNA gene sequencing. Batch 10 mL cultures of each strain was cultured in triplicate in the growth medium of Strobel (13) in the presence and absence of 50 µg/L cyanocobalamin (Sigma-Aldrich, St. Louis, MO, USA), and incubated at 39°C for 48 h. Optical density measurements at 600 nm were taken using a Spectronic 200 spectrophotometer (Thermo Fisher Scientific, Waltham, MA, USA) to monitor growth. Fermentation end-products were quantified from culture supernatants by gas chromatography, as previously described (28).

### 16S rRNA phylogeny

Near full-length 16S rRNA sequences of screened Hungate1000 strains (15) were aligned using MUSCLE v5.0 (29), and a maximum-likelihood tree was generated using the default settings in MEGA X (30). The resulting tree was further visualized using iTOL v4 (31).

### Genome sequencing and analyses

The complete genome of *X. ruminicola* KHP1 was sequenced using a hybrid 2 × 150 bp short read (MGI Tech, Shenzhen, China) and long read (Oxford Nanopore Technologies, Oxford, UK) sequencing strategy and assembled, as previously described (32). Gene calling and annotation were carried out using the Prokaryotic Genome Annotation Pipeline (PGAP) v5.0 (33). Genomic analyses of Hungate1000 genomes (15) were carried out using the Integrated Microbial Genomes server (IMG/MER) (34). The circular genome map was generated using GenoVi v0.2.16 (35). Linear gene maps were generated using GenomeDiagram (36) in Biopython (37). The KHP1 genome was scanned for cobalamin riboswitch families: “cobalamin,” “adoCbl,” and “adoCbl-variant,” using Riboswitch Scanner (38). Propionate pathway gene homologs were determined based on BLASTP (39) homology of encoded proteins to those identified in *X. ruminicola* 23^T^ in Zhang et al. (16). Gene functional assignments to the Clusters of Orthologous Genes (COGs) database (40) were made using eggNOG-mapper v2.0 (41). Average nucleotide identity (ANI) distances were calculated using fastANI v1.32 (42) under default settings.

### Comparative transcriptomics/proteomics experiment of cobalamin-induced propionate production

To investigate the genes and proteins that are differentially regulated during cobalamin induction of the propionate pathway in KHP1, 100 mL cultures were grown in 250 mL serum bottles (5% inoculum size) in the medium described in Strobel (13) in the presence and absence of 50 µg/L cobalamin supplementation (*n* = 6 per treatment) to generate samples for transcriptome and proteome analyses. Immediately after inoculation, and at two-hourly time points thereafter, a 1 mL sample was anaerobically taken from each culture and used to measure optical density (600 nm), and fermentation end-product concentrations as previously described (28). Cultures were harvested after 10 h when cultures were approximately in late log phase growth by flash-freezing in liquid nitrogen, followed by storage at −80°C until samples were required for transcriptome and proteome analyses.

### RNA-seq analyses

Total RNA was extracted from approximately 4 g of frozen KHP1 cultures using a modified acid phenol/chloroform procedure of mechanically lysed cells by bead-beating, as previously described (43). RNA was DNase-treated using a Turbo DNAse kit (Thermo Fisher Scientific) and was purified using the MEGAclear transcription clean-up kit (Invitrogen, Waltham, MA, USA) according to the manufacturer’s instructions. RNA samples were quantified by Qubit (Invitrogen) and integrity assessed using a BioAnalyzer 2100 with the RNA 6000 Nano kit (Agilent Technologies, Santa Clara, CA, USA). The RNA samples were sequenced by Novogene (Beijing, China), with ribosomal RNA depletion using the RiboZERO Magnetic kit (Illumina), and library preparation using the NEBNext Ultra II Directional RNA Library Prep kit (Illumina). Libraries were sequenced on an Illumina NovaSeq 6000 instrument (2 × 150 bp paired-end, ~2 Gb per sample).

Raw transcriptome sequence reads were trimmed of adapter sequences (default settings) and quality-filtered (-q 20) using cutadapt v3.3 (44). Filtered reads were aligned to the complete KHP1 genome using HISAT2 v2.2.1 (45) under default settings. SAM alignment files were converted to BAM format using SAMtools v1.11 (46), and read alignment counts were extracted using FeatureCounts v2.0.1 (47). The resulting matrix was input into R v4.1.1 (48), and read counts were log_2_-transformed. Differentially expressed genes (DEGs) were identified between treatments using the DESeq2 package v1.32.0 (49), with significance defined using FDR-adjusted (50) *P* < 0.05 and |log_2_ fold change| ≥ 1 when analyzing all genes, with the lower threshold of |log_2_ fold change| ≥ 0.5 applied when analyzing propionate pathway genes. Principal component analysis (PCA) was carried out on log_2_-transformed read counts of each gene using the “prcomp” function, and plotted using ggfortify v0.4.11 (51).

### Proteome analyses

An aliquot of 500 µL of each thawed culture was briefly vortexed in 250 µL of lysis buffer (30 mM dithiothreitol, 150 mM Tris-HCl [pH 8], 0.3% [vol/vol] Triton X-100, 12% [wt/vol] SDS) and kept on ice for 30 min. Lysis was then performed with ≤106 µm glass beads using a FastPrep 24 classic grinder (MP Biomedicals, Santa Ana, CA, USA) for three 60 s cycles at 4.0 m/s. Samples were centrifuged at 16,000 × *g* at 4°C, and lysate was carefully removed. A 40–50 µg aliquot of protein from each sample was prepared in SDS buffer, heated in a 99°C water bath for 5 min and analyzed by SDS-PAGE using Any-kD mini-PROTEAN TGX stain-free gels (Bio-Rad, Hercules, CA, USA) in a 2 min run for sample clean-up, before staining with Coomassie Blue R-250. Visible bands were excised from the gel and destained, reduced, alkylated, digested, and the resulting peptides were extracted and desalted using the OASIS HLB µElution plate (52) following the manufacturers’ instructions. Peptides were analyzed by nano-LC-MS/MS using a Q-Exactive hybrid quadrupole Orbitrap MS (Thermo Fisher Scientific) as previously described (53). Raw mass spectrometry files were analyzed using the MaxQuant platform (54) and proteins were identified and quantified using the MaxLFQ algorithm (55). Data were searched against predicted amino acid sequences from the complete KHP1 genome. ProteinGroups files from MaxQuant were further processed and analyzed in Perseus (56), with quality filtering performed under default settings. Label-free quantification intensities of filtered proteins were logarithmically normalized (log_2_), and missing data were imputed based on the normal distribution. Differentially abundant proteins were identified by two-sample *t* tests, with significance defined using FDR-adjusted (permutation-based method with 250 randomizations) *P* < 0.05 and |log_2_ fold change| ≥ 1 when analyzing all proteins, with the lower threshold of |log_2_ fold change| ≥ 0.5 applied when analyzing propionate pathway proteins. PCA was carried out as for the transcriptome data using the log_2_-transformed, imputed data as input.

## RESULTS

### Cobalamin-dependent propionate production prevalent among ruminal *Xylanibacter* and related strains

**Fig 1:**
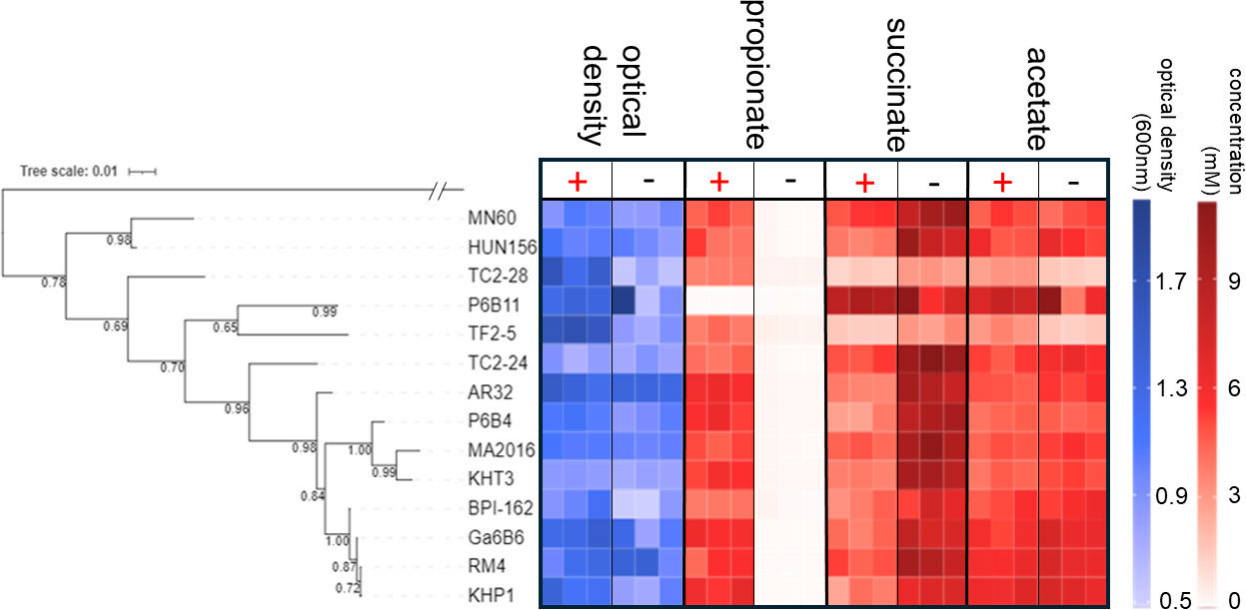
Cobalamin-dependent propionate production of ruminal *Xylanibacter* and related strains. A phylogenetic maximum-likelihood tree generated from alignments of near full-length 16S rRNA genes, with sequence from *Butyrivibrio proteoclasticus* B316^T^ as an outgroup. Tree is shown alongside OD_600_ optical density (blue heatmap) and propionate, succinate, and acetate concentrations (red heatmap) of cultures grown for 48 h in cobalamin-supplemented (+) and non-supplemented (−) media. Heatmap squares within each category represent values for each of the three replicate cultures.

To assess the effect of cobalamin supplementation on the growth and fermentation of ruminal *Xylanibacter* and related strains previously classified via 16S rRNA phylogeny as “*Prevotella* 1” (27), 14 strains from the Hungate1000 culture collection (15; Table S1) were cultured with and without cobalamin supplementation (13). The optical density and fermentation end-product concentrations after 48 h of growth showed that all strains produced propionate in a cobalamin-dependent manner, apart from *X. brevis* P6B11, which accumulated greater succinate and acetate concentrations instead (Fig. 1). None of the strains required cobalamin supplementation for growth, although some, such as TF2-5 and TC2-28, grew to greater optical densities when it was supplied (Student’s *t* test; *P* ≤ 0.01). Both TF2-5 and TC2-28 also appeared to produce less succinate and acetate than other strains (Fig. 1).

### Detection of propionate pathway genes and cobalamin riboswitches in the complete KHP1 genome

To characterize cobalamin regulation of propionate production via the succinate pathway in *Xylanibacter*, *X. ruminicola* KHP1 was selected for further analyses. KHP1 exhibited cobalamin-dependent propionate production (average of 6.0 ± 0.6 mM after 48 h) and cultures grew well and to similar optical densities with and without cobalamin supplementation (Fig. 1). KHP1 also shares close values of 99.5% 16S rRNA gene sequence identity and 97.4% genome-wide ANI with strain 23^T^ (Table S1).

**Fig 2:**
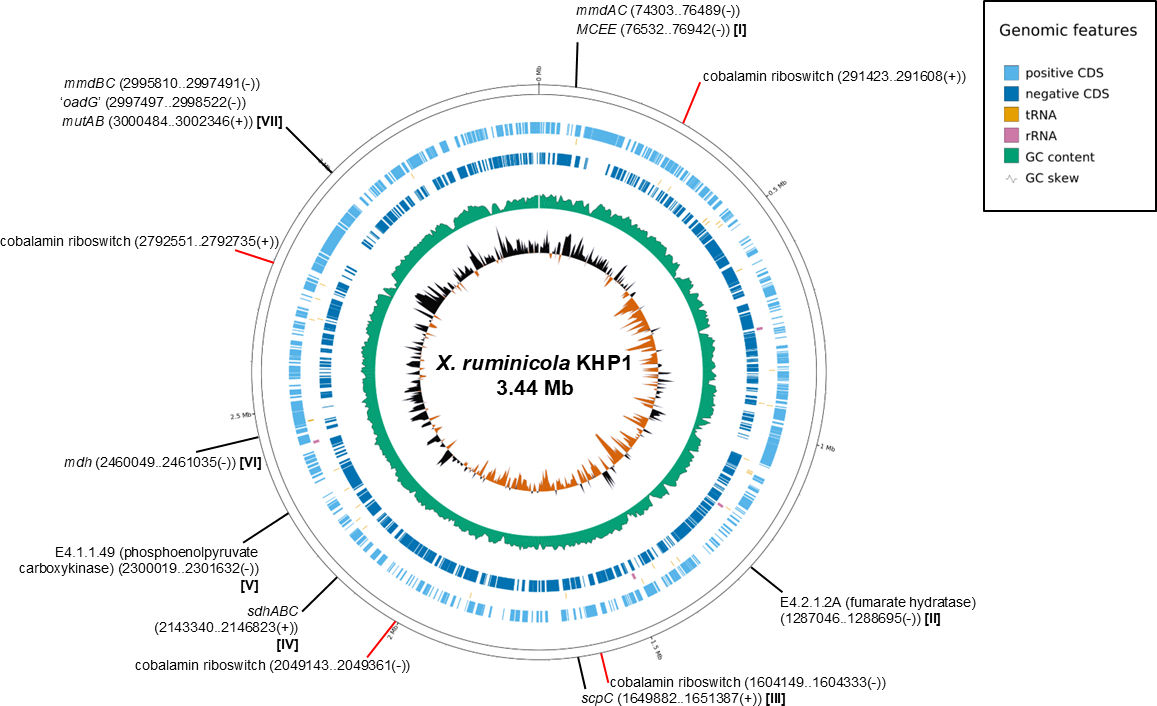
Distribution of propionate pathway genes in the KHP1 genome. Genome map of KHP1 showing the positions of homologs of propionate pathway genes, and putative cobalamin riboswitches.

The originally available draft genome of KHP1 by Seshadri et al. (15) was incomplete and comprised of six contigs. Hence, we generated the complete genome sequence of KHP1, which was a single circular 3.4 Mb contig, with G + C content of 47.8% (Table S2). Homologs of all proteins of the propionate pathway described for *X. ruminicola* 23^T^ (16) were present in the KHP1 genome (Table S3). Moreover, for all strains screened for cobalamin-induced propionate production above, all gene homologs were also present in their genomes (Table S4). However, it was noted that all propionate-producing strains displayed a conserved arrangement of methylmalonyl-CoA decarboxylase subunit genes (*mmdB* and *mmdC*) positioned closely upstream but in the antisense orientation to methylmalonyl-CoA mutase subunit genes (*mutA* and *mutB*). In contrast, in P6B11 the decarboxylase genes were co-located on a separate contig to the methylmalonyl-CoA mutase genes, which were also further apart from each other (Fig. S1). As for strain 23^T^, the KHP1 propionate pathway genes were dispersed at several loci in the genome (Fig. 2), only two of which encoded subunits for more than one pathway enzyme: at locus VII, *mutA* and *mutB*, were near but antisense to *mmdB* and *mmdC*. An additional gene annotated as “oxaloacetate decarboxylase gamma subunit” (designated “*oadG*”) was positioned in a putative operon with *mmdB* and *mmdC*, where the gamma subunit of oxaloacetate decarboxylase has structural analogy to the delta subunit of methylmalonyl-CoA decarboxylase (17). An exact match of “OadG” was also encoded by an identically positioned gene in *X. ruminicola* 23^T^ (PRU_RS08500). At locus I, a gene for methylmalonyl-CoA epimerase (*MCEE*) was located in a putative operon downstream of *mmdA* and *mmdC*, which were separated by a gene for a short 50 aa hypothetical protein (Fig. 2).

The KHP1 genome was also scanned for cobalamin riboswitches, given their prominent role in the regulation of cobalamin-mediated processes in other bacteria (21). Four cobalamin riboswitches were detected, though none were co-located with any of the propionate pathway genes (Fig. 2; Table S5).

### Transcriptome and proteome analysis of cobalamin-supplemented KHP1 cultures

**Fig 3:**
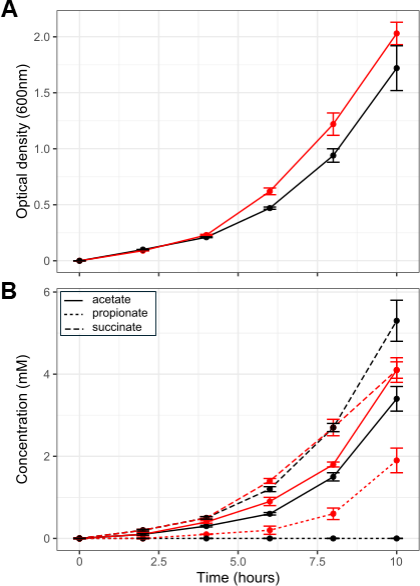
Optical density (**A**) and fermentation end-product (**B**) formation of KHP1 cultures over 10 h of growth. Error bars denote one SEM (*n* = 6) of cultures grown in cobalamin-supplemented (red) and non-supplemented (black) media.

To identify the genes and proteins that are upregulated during cobalamin induction of the propionate pathway in KHP1, cultures grown with and without cobalamin supplementation were used for transcriptome and proteome analyses. Cobalamin-supplemented cultures grew to significantly higher optical densities after 6 and 8 h (Student’s *t* test; *P* = 0.002 and 0.03, respectively), but not when harvested after 10 h (Student’s *t* test; *P* > 0.05) (Fig. 3A). Acetate production was also greater in cobalamin-supplemented cultures at 6 h (Student’s *t* test; *P* = 0.01), but not at any other time points (Student’s *t* test; *P* > 0.05) (Fig. 3B). Propionate was detected only in cobalamin-supplemented cultures and coincided with lower succinate concentrations when harvested after 10 h (Fig. 3B) trending toward significance (Student’s *t* test; *P* = 0.07).

**Fig 4:**
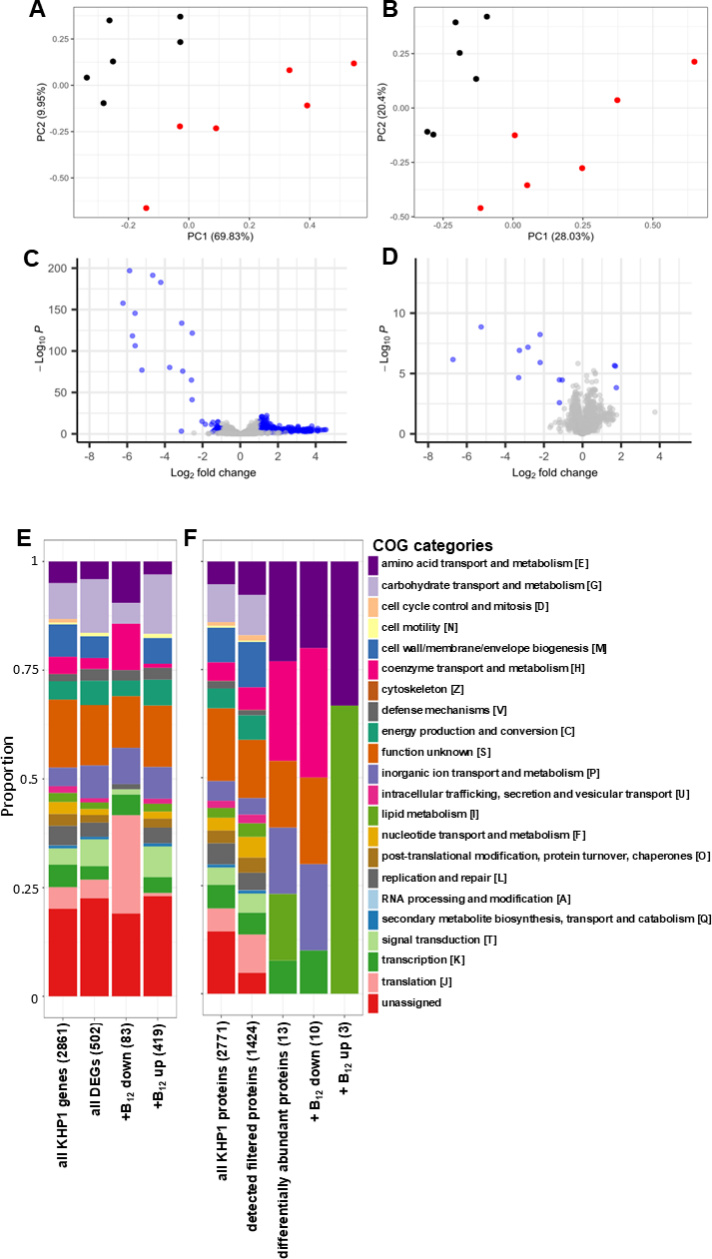
Cobalamin-induced shifts in the KHP1 transcriptome and proteome. PCA plots of (**A**) transcriptome data, and (**B**) proteome data from cobalamin-supplemented (red) and non-supplemented (black) cultures. Volcano plots of differentially expressed (**C**) transcripts and (**D**) proteins. Differentially expressed genes and proteins (FDR-adjusted *P* < 0.05, |log_2_ fold change| ≥ 1) are shown in blue. Positive log_2_ fold changes represent an increase in abundance in the presence of cobalamin. Stacked bar plots showing relative abundances of (**E**) transcripts and (**F**) proteins by COG category.

RNA-seq resulted in an average of 8,178,217 ± 976,705 quality-filtered (*Q* > 20) reads per sample, which aligned to the KHP1 genome at an average of 98.7 ± 0.4% of reads per sample. PCA plots of log_2_-transformed read counts showed clear separation of transcriptome profiles between cobalamin treatments (Fig. 4A). A total of 502 DEGs were identified, of which 419 were upregulated, and 83 were downregulated as a result of cobalamin supplementation (Table S6). Despite far fewer genes being downregulated, these differences were generally more highly statistically significant, and showed greater differences in transcript abundance (Fig. 4C). Many DEGs were not assigned to any COG category (22.4%) or to the category “function unknown [S]” (13.7%). Of DEGs assigned to functionally descriptive categories, the most abundant were “carbohydrate metabolism and transport [G]” (12.3%), “inorganic ion transport and metabolism [P]” (7.5%), and “signal transduction [T]” (6.1%). Categories enriched for genes downregulated by cobalamin supplementation included “translation [J]” (22.6%), unassigned to any COG (19%), and assigned to “function unknown [S]” (11.9%). Of the categories assigned to genes upregulated by cobalamin supplementation, the most abundant were unassigned to any COG (23%), assigned as “function unknown [S]” (14.1%), and “carbohydrate metabolism and transport [G]” (13.7%) (Fig. 4E). Many of the most highly differentially expressed genes were closely located to the putative cobalamin riboswitches (Fig. S2). This most notably included a putative operon of six genes immediately downstream of the cobalamin riboswitch identified at position 291,423..291,608 containing four genes annotated as cobalamin biosynthesis enzymes and two annotated as TonB-dependent transporters, which have known roles in cobalamin transport in other bacteria (57). Transcripts encoded by these six genes were among the seven most highly differentially expressed genes of the data set (Fig. S2A).

Proteome analyses of the samples using untargeted LC-MS/MS identified a total of 1,853 proteins, of which, 1,424 passed downstream quality filtering. PCA plots also showed clear separation of the samples between each treatment (Fig. 4B). Only 13 differentially abundant proteins were identified, 3 with greater abundances in the presence and 10 in the absence of cobalamin supplementation (Fig. 4D; Table S7). As for the transcriptome, many of the differentially expressed proteins were closely located to the putative cobalamin riboswitches (Fig. S2).

### Transcriptome and proteome analysis of propionate pathway genes

**Fig 5:**
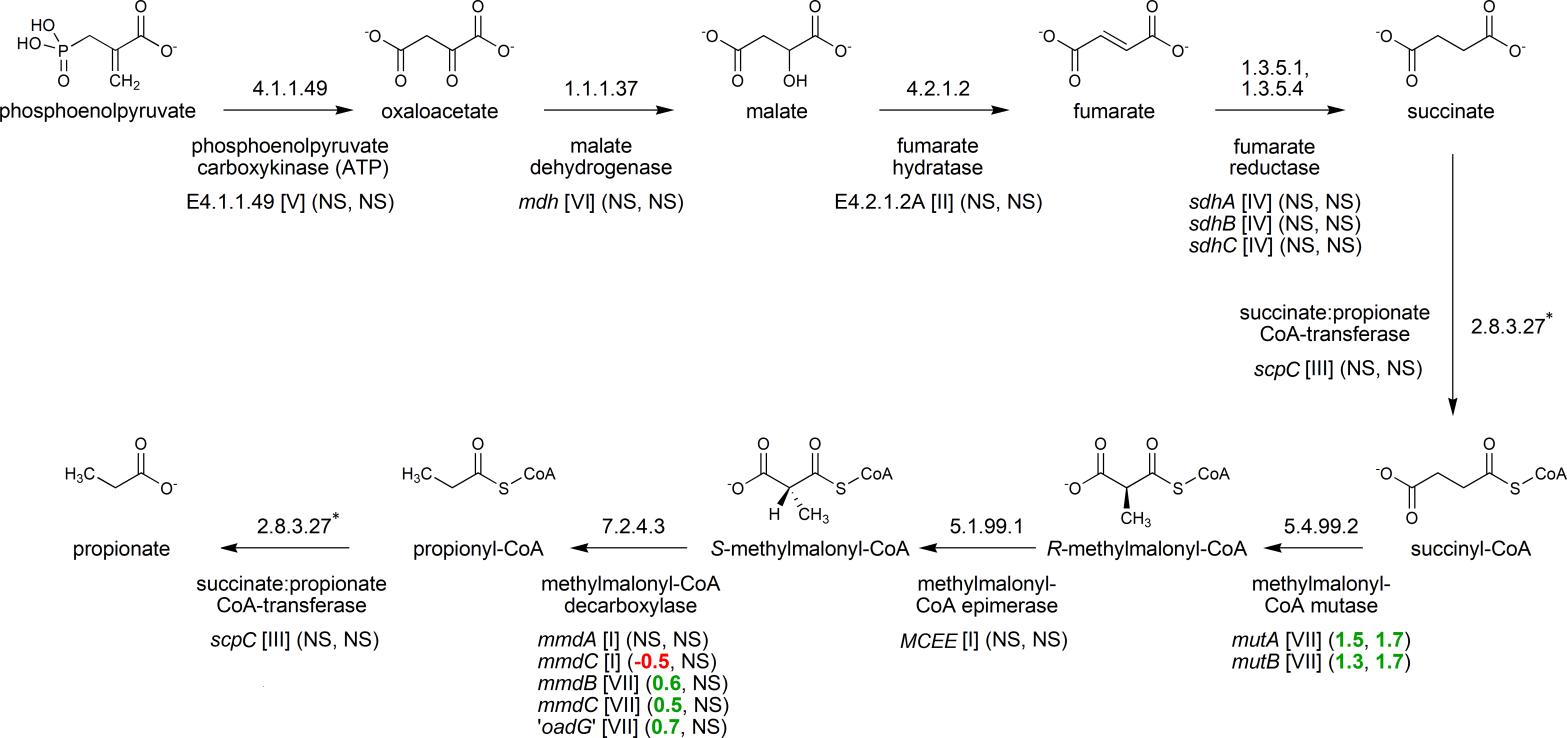
Impact of cobalamin on the expression of succinate pathway transcripts and proteins. Numbers in brackets next to each gene represent log_2_ fold changes of transcript (left) and protein (right) abundances between treatments. In both instances, positive (green) values represent increased abundance in the cobalamin-supplemented compared to non-supplemented medium. NS = not significant (FDR-adjusted *P* > 0.05). *, coupled reaction.

A summary of the impact of cobalamin on the expression of transcripts and proteins of genes involved in propionate production is shown in Fig. 5. Transcripts of *mutA* and *mutB* were both upregulated by cobalamin supplementation. The co-located *mmdB*, *mmdC*, and “*oadG”* genes were also upregulated, though only marginally (log_2_ fold change <0.8) (Fig. S3). At the proteome level, the only differentially abundant propionate pathway proteins were both subunits of methylmalonyl-CoA mutase, which were more abundant in the presence of cobalamin supplementation. Thus, *mutA* and *mutB* were the only propionate pathway genes that consistently showed higher expression at both transcriptome and proteome levels in cobalamin-supplemented cultures, though these expression changes were all relatively minor (log_2_ fold change 1.3–1.7) (Fig. 5).

Enzymes involved in the terminal conversion of CoA-thioesters to their respective SCFAs are known to characteristically exhibit broad substrate specificity (7, 58), thus their gene and protein annotations may not necessarily represent the predominant substrate for the enzyme. The KHP1 genome contained co-located genes annotated as phosphate butyryltransferase (EC 2.3.1.19) (J4031_09245; 2220507..2221343) and butyrate kinase (EC 2.7.2.7) (J4031_09250; 2221343..2222413), despite not producing any butyrate. Both genes were upregulated by cobalamin supplementation (Table S5). These genes were the first two in a putative operon including genes for two HAMP domain-containing proteins (59), a TlpA family disulfide reductase and a tetracopeptide repeat-containing protein, all of which were upregulated by cobalamin supplementation. However, the phosphate butyryltransferase was not differentially abundant within the proteome, and the butyrate kinase protein was removed during quality-filtering and could not be analyzed.

Although separate from the pathway of propionate production itself, given the involvement of the ferredoxin-NAD^+^ oxidoreductase (Na^+^ transporting) (EC 7.2.1.2) Rnf complex in recycling reduced electron carriers to enable fermentation via the succinate pathway in *X. ruminicola* (16), we also assessed whether the encoding genes of Rnf were differentially expressed between cobalamin treatments. However, all six co-located genes of the complex (CP071890; 557338..562564) were not significantly differentially expressed at the transcriptome level, and only the RnfC subunit met quantity and quality thresholds for analysis in the proteome data set, which was also not significantly differentially abundant (Tables S6 and S7).

Given that the only propionate pathway genes upregulated at both transcript and protein levels were *mutA* and *mutB*, potential regulatory mechanisms were investigated by searching the KHP1 genome for genes encoding proteins known to interact with methylmalonyl-CoA mutase (Table S8). The methylmalonyl-CoA mutase-associated GTPase, MeaB, has been shown to be part of the methylmalonyl-CoA mutase complex, playing an essential role of preventing irreversible inactivation of the complex (60). One MeaB homolog was identified via the KHP1 genome (J4031_05445, 1227141..1228241; 49% aa identity). However, there were no significant differences between treatments at the transcriptome level, nor of the corresponding protein (QVJ81811) at the proteome level. We also examined the KHP1 genome for genes encoding cobalamin adenosyltransferase (EC 2.5.1.17), the enzyme responsible for the conversion of cobalamin to the bioactive form adenosylcobalamin, and its delivery to the methylmalonyl-CoA mutase complex (61). The KHP1 genome possessed a gene homolog for PduO-type cobalamin adenosyltransferase with greatest homology to the PduO-type adenosyltransferase of *Cupriavidus metallidurans* (J4031_10040, 2417760..2418233; 41% aa identity) (62), which was highly significantly upregulated at the transcriptome level (log_2_FC = 1.56, FDR-corrected *P* = 2.4 × 10^−4^), though the corresponding protein (QVJ80035) was not detected in the proteome data. Genes for neither MeaB nor the PduO adenosyltransferase homologs were located near any of the propionate pathway genes, nor the putative cobalamin riboswitches.

## DISCUSSION

The aim of this study was to experimentally assess the prevalence of cobalamin induction of propionate production in ruminal *Xylanibacter in vitro*, and the impact of cobalamin supplementation on propionate pathway expression in *X. ruminicola* KHP1. Screening of a selection of *Xylanibacter* and closely related strains from the Hungate1000 collection confirmed widespread cobalamin-dependent production of propionate across the group. This is unsurprising given all strains examined contained all homologs of genes in the propionate production pathway (16). Moreover, although we did not observe *X. brevis* P6B11 to produce propionate under the growth conditions of this study, we have found it to produce small amounts of propionate in cobalamin-supplemented rich media containing 30% (vol/vol) rumen fluid. The *X. brevis* type strain also produces small amounts of propionate in culture (16). *In silico* analyses of the complete KHP1 genome identified four putative cobalamin riboswitches, two of which were located immediately upstream of gene(s) with annotations suggestive of roles in cobalamin transport and/or biosynthesis. However, these were not located near any of the propionate pathway genes, which were positioned at numerous loci in the genome. Transcriptome and proteome analyses of KHP1 cultures grown with and without supplemented cobalamin revealed differential transcription of some pathway genes encoding subunits of methylmalonyl-CoA mutase and methylmalonyl-CoA decarboxylase. However, only the cobalamin-dependent methylmalonyl-CoA mutase genes were also differentially abundant at the proteome level, and the magnitude of differential expression at both levels was relatively minor. These results suggest constitutive expression of the propionate pathway irrespective of cobalamin supplementation, and that activation of methylmalonyl-CoA mutase through direct binding of its adenosylcobalamin cofactor may be the pivotal step in the pathway to enable propionate production.

The apparent constitutive expression of propionate pathway enzymes regardless of cobalamin supplementation might be explained by the ubiquity of cobalamin in the rumen. Cobalamin is exclusively synthesised by bacteria and requires cobalt which is generally supplied through the diet. The rumen is the central location of cobalamin biosynthesis in ruminants (63), and therefore under normal physiological conditions with sufficient cobalt (64) the rumen is rarely likely to experience cobalamin limitation. This appears particularly true for former ruminal *Prevotella*, as the abundance of this group has been shown to be positively correlated with ruminal cobalamin concentrations (65). Moreover, the identification of a number of cobalamin biosynthesis genes in the KHP1 genome suggests *X. ruminicola* may be capable of partial synthesis of cobalamin via the salvage pathway (66), and would thereby only require cobalamin precursors to be able to satisfy their cobalamin requirement. Constitutive expression of the propionate pathway in *X. ruminicola* may therefore be of adaptive value in the rumen, and there may have been little evolutionary pressure for expression of the propionate pathway to be repressed in the absence of cobalamin. Additionally, under conditions where propionate production was suppressed, given the broad substrate specificity of terminal enzymes in the propionate pathway (7), they may still be used in alternative metabolic pathways of importance to the cell.

The mechanism(s) by which genes for methylmalonyl-CoA mutase, methylmalonyl-CoA decarboxylase, and cobalamin adenosyltransferase were upregulated in the presence of cobalamin supplementation is not known but does not appear to directly involve cobalamin riboswitches, as the four detected in the KHP1 genome were not in close proximity to any of the genes encoding propionate pathway enzymes nor the methylmalonyl-CoA mutase accessory proteins. The relatively minor effects observed might potentially occur via direct or indirect routes. Cobalamin supplementation of KHP1 cultures led to the differential expression of a large proportion of the transcriptome, with approximately 20% of KHP1 genes affected. In contrast, only 0.9% of proteins were differentially expressed, indicating the extent of impact on general cellular function is likely less than that observed on the transcriptome, although it should also be noted that only approximately half of the proteins from open reading frames predicted in the KHP1 genome passed filtering criteria and could be analyzed in the final proteome data set. There is increasing awareness of the roles of cobalamin as a cofactor of regulatory proteins (67, 68). Cobalamin supplementation led to the differential expression of genes predicted to encode regulatory proteins, though while none were detected as differentially abundant at the proteome level, their typically low abundance can prevent their detection in proteome data (69). It is also possible that other post-translational modifications may have contributed to these effects (70). Given the direct effects of propionate observed toward gene expression of cells of other organisms (71–73), some of the observed effects may be mediated by propionate itself.

Analyses of genes encoding subunits of the sodium ion-translocating methylmalonyl-CoA decarboxylase responsible for the decarboxylation of *S*-methylmalonyl-CoA to propionyl-CoA were of particular interest, as this was the only succinate pathway enzyme in which encoding genes were present in two genomic loci, contrasting the genomic arrangement in characterized methylmalonyl-CoA decarboxylases of *V. parvula* and *Propionigenium modestum*, where all genes are arranged in a single operon (74). Despite this, it was also noteworthy that genes at both loci were not co-ordinately differentially expressed by cobalamin supplementation. The *mmdB* and *mmdC* genes at the *mutAB* locus were slightly upregulated, whereas the *mmdA* and *mmdC* genes located at the *MCEE* locus were either not differentially expressed or slightly downregulated. Moreover, at the *mutAB* locus an additional subunit designated as an “oxaloacetate decarboxylase gamma subunit” was identified on the basis of updated annotations. As the delta subunit of methylmalonyl-CoA decarboxylase is structurally analogous to the gamma subunit of oxaloacetate decarboxylase (17), this very likely constitutes the previously missing *mmdD* gene (16), thereby solving the genomic prediction of a complete functional complex in *X. ruminicola*, though further enzymatic characterization is necessary to confirm this.

The final step of the pathway in which propionyl-CoA is converted to propionate via the succinate pathway has been traditionally considered to be carried out by succinate:propionate CoA-transferase, coupled with the earlier conversion of succinate to succinyl-CoA (75). More broadly, during fermentation, the production of SCFAs from their respective CoA thioesters can occur either by direct CoA-transfer, or in two steps via an acyl-phosphate intermediate (7). It was therefore noteworthy that, despite not producing butyrate, KHP1 possessed genes for a putative phosphate butyryltransferase and butyrate kinase, both of which were upregulated by cobalamin supplementation at the transcriptome level. As characterized enzymes responsible for these steps often act on multiple substrates (58, 76, 77), the upregulation of these genes in the cobalamin-supplemented cultures when propionate was being produced suggests these enzymes may have phosphate propanoyltransferase (EC 2.3.1.8, 2.3.1.222)/propionate kinase (EC 2.7.2.1, 2.7.2.15) activity and contribute to propionate production in *X. ruminicola* via propionyl phosphate. Future biochemical characterization of these gene products will shed further light on their potential involvement in propionate production and enable annotation of these genes to be revisited.

It is noted that the growth medium used in this study contains small amounts of cobalamin through the inclusion of tryptone, which was estimated to supply less than 0.45 µg/L in Strobel (13). While any cobalamin present in the absence of cobalamin supplementation was insufficient to induce propionate production, we cannot exclude the possibility that this could have induced the expression of propionate pathway genes, hence their apparent constitutive expression under both cobalamin supplementation and non-supplementation treatments. However, we argue that if such trace levels of cobalamin induced expression of the propionate pathway genes, this would seem counterintuitive when cobalamin concentrations are insufficient for post-translational activation of propionate production. Moreover, the clear differential expression of genes under apparent control of putative cobalamin riboswitches provides further indication of the likely negligible nature of any trace cobalamin present in the growth medium used. Future confirmation of these results using medium containing a cobalamin-free nitrogen source would provide further insight into the constitutive expression of the propionate pathway.

In summary, our results show methylmalonyl-CoA mutase as the only propionate pathway enzyme that was consistently upregulated by cobalamin supplementation at both transcriptome and proteome levels under *in vitro* conditions. All effects of cobalamin supplementation on propionate pathway genes were relatively minor in magnitude, suggesting the lack of any major regulatory mechanism induced by cobalamin supplementation activating the pathway at transcriptome or proteome levels. Instead, the cobalamin-dependent induction of propionate production appears to be primarily controlled post-translationally by the interaction of methylmalonyl-CoA mutase with its adenosylcobalamin cofactor. The precise mechanisms by which cobalamin supplementation affected the expression of methylmalonyl-CoA mutase genes and proteins will be the subject of further investigation, as well as whether genes annotated as phosphate butyryltransferase and butyrate kinase contribute toward propionate production in *X. ruminicola* as suggested by our transcriptome data.

## Data Availability

The KHP1 genome has been deposited in GenBank under accession CP071890, BioProject PRJNA715253. Raw RNA-seq reads have been deposited in the Sequence Read Archive (SRA) under BioProject PRJNA1116615. Raw proteomics data have been deposited to the ProteomeXchange consortium (http://proteomecentral.proteomexchange.org) via the PRIDE partner repository (78) with the data set identifier PXD053273.

## SUPPLEMENTAL MATERIAL

Supplemental Tables and Figures msystems.00864-24-s0001.docx Tables S1 to S8; Fig. S1 to S3.

ASM does not own the copyrights to Supplemental Material that may be linked to, or accessed through, an article. The authors have granted ASM a non-exclusive, world-wide license to publish the Supplemental Material files. Please contact the corresponding author directly for reuse.
