# Supplementary material for: Transcriptomic and proteomic changes associated with cobalamin-dependent propionate production by the rumen bacterium Xylanibacter ruminicola
Source: mSystems. 2024 Oct 29;9(11):e00864-24. doi: 10.1128/msystems.00864-24 (PMC11575231; doi:10.1128/msystems.00864-24)
Supplement: Supplemental Tables and Figures — Tables S1 to S8; Fig. S1 to S3. [file msystems.00864-24-s0001.docx]

**Table S1. Reference Hungate1000 strains screened in this study**

| Strain | Strain host, country | % ID to *X. ruminicola* 23^T^ 16S rRNA gene | % ANI to *X. ruminicola* 23^T^ genome | GTDB taxonomy^1^  (genus, species) |
| --- | --- | --- | --- | --- |
| P6B11 | Cow, New Zealand | 92.3 (98.7)^2^ | 79.3 (97.5)^2^ | g__*Prevotella*; s__*Prevotella brevis* |
| BPI-162 | Sheep, Japan | 98.7 | 96.95 | g__*Prevotella*; s__*Prevotella ruminicola* |
| KHP1 | Cow, New Zealand | 99.5 | 97.4 | g__*Prevotella*; s__*Prevotella ruminicola* |
| RM4 | Cow, Japan | 97.1 | 97.6 | g__*Prevotella*; s__*Prevotella ruminicola* |
| KHT3 | Cow, New Zealand | 95.9 | 82.5 | g__*Prevotella*; s__*Prevotella ruminicola_A* |
| Ga6B6 | Cow, New Zealand | 99.0 | 94.3 | g__*Prevotella*; s__*Prevotella ruminicola*_B |
| MA2016 | Cow, New Zealand | 96.2 | 85.5 | g__*Prevotella*; s__*Prevotella* sp000619825 |
| P6B4 | Cow, New Zealand | 96.3 | 84.9 | g__*Prevotella*; s__*Prevotella* sp000702825 |
| TC2-28 | Cow, Sweden | 91.9 | 78.3 | g__*Prevotella*; s__*Prevotella* sp900107705 |
| AR32 | Sheep, Australia | 96.7 | 80.8 | g__*Prevotella*; s__*Prevotella* sp900109055 |
| TC2-24 | Cow, Sweden | 92.6 | 78.8 | g__*Prevotella*; s__*Prevotella* sp900110895 |
| TF2-5 | Cow, Sweden | 91.8 | n/a^3^ | g__*Prevotella*; s__*Prevotella* sp900115435 |
| HUN156 | Cow, New Zealand | 90.7 | 79.3 | g__*Prevotella*; s__*Prevotella* sp900119285 |
| MN60 | Sheep, Japan | 89.4 | 79.4 | g__*Prevotella*; s__*Prevotella* sp900199555 |

^1^ GTDB version 09-RS220. ^2^ Similarities to *X. brevis* GA33^T^ are shown in brackets.
^3^ Alignment fractions of genomes below the default 20% threshold and therefore not computed.

**Table S2. Genome statistics of *Xylanibacter ruminicola* KHP1**

| Parameter | Data for strain KHP1^1^ |
| --- | --- |
| BioProject accession no. | [PRJNA715253](https://www.ncbi.nlm.nih.gov/bioproject/PRJNA715253) |
| BioSample accession no. | [SAMN18341379](https://www.ncbi.nlm.nih.gov/biosample/SAMN18341379) |
| GenBank accession no. | [CP071890](https://www.ncbi.nlm.nih.gov/nuccore/CP071890.1) |
| SRA accession no. (ONT) | [SRR16277835](https://dataview.ncbi.nlm.nih.gov/object/SRR16277835) |
| SRA accession no. (MGISEQ) | [SRR16277834](https://dataview.ncbi.nlm.nih.gov/object/SRR16277834) |
| No. of raw ONT reads | 617,812 |
| No. of filtered ONT reads | 577,710 |
| *N*_50_ of filtered ONT reads (bp) | 5,042 |
| No. of raw MGISEQ reads | 6,862,268 |
| No. of filtered MGISEQ reads | 6,854,260 |
| Genome size (Mb) | 3.44 |
| No. of contigs | 1 |
| Sequencing coverage (×) | 514 |
| G+C content (%) | 47.76 |
|  |  |
| Genes (total) | 2,861 |
| CDSs (total) | 2,793 |
| CDSs (with protein) | 2,771 |
| Genes (RNA) | 68 |
| rRNA genes (5S, 16S, 23S) | 4, 4, 4 |
| tRNA genes | 54 |
| Noncoding RNA genes | 2 |
| Pseudogenes | 22 |

^1^ Data are based on Prokaryotic Genome Annotation Pipeline (PGAP) annotations.

**Table S3. Homologues of propionate pathway genes in the KHP1 genome**

| Enzymatic step | EC number | Enzyme name | Enzyme subunit | Protein ID in 23^T 1^ | KHP1 gene homologue | KHP1 protein homologue | % aa identity |
| --- | --- | --- | --- | --- | --- | --- | --- |
| phosphoenolpyruvate 🡪 oxaloacetate | 4.1.1.49 | phosphoenolpyruvate carboxykinase (ATP) | E4.1.1.49 | PRU_2279 | J4031_09580 | QVJ79957.1 | 100 |
| oxaloacetate 🡪 malate | 1.1.1.37 | malate dehydrogenase | Mdh | PRU_2158 | J4031_10175 | QVJ80061.1 | 99.1 |
| malate 🡪 fumarate | 4.2.1.2 | fumarate hydratase | E4.2.1.2A | PRU_0119 | J4031_05685 | QVJ81858.1 | 99.6 |
| fumarate 🡪 succinate | 1.3.5.1, 1.3.5.4 | fumarate reductase | SdhA  SdhB  SdhC | PRU_2432  PRU_2431  PRU_2433 | J4031_08890  J4031_08895  J4031_08885 | QVJ79830.1  QVJ79831.1  QVJ79829.1 | 98.6  99.6  99.6 |
| succinate 🡪 succinyl- CoA | 2.8.3.27 | succinate:propionate CoA-transferase | ScpC | PRU_2762 | J4031_07180 | QVJ82124.1 | 99.6 |
| succinyl-CoA 🡪 *R*-methylmalonyl-CoA | 5.4.99.2 | methylmalonyl-CoA mutase | MutA | PRU_1640 | J4031_12560 | QVJ80503.1 | 99.4 |
|  |  |  | MutB | PRU_1639 | J4031_12565 | QVJ80504.1 | 99.9 |
| *R*-methylmalonyl-CoA 🡪 *S*-methylmalonyl-CoA | 5.1.99.1 | methylmalonyl-CoA epimerase | MCEE | PRU_1230 | J4031_00330 | QVJ80881.1 | 100 |
| *S*-methylmalonyl-CoA 🡪 propionyl-CoA | 7.2.4.3 | methylmalonyl-CoA decarboxylase | MmdA | PRU_1231 | J4031_00325 | QVJ80880.1 | 99.0 |
|  |  |  | MmdB | PRU_1644 | J4031_12540 | QVJ80499.1 | 100 |
|  |  |  | MmdC | PRU_1232  PRU_1643 | J4031_00315 J4031_12545 | QVJ80878.1  QVJ80500.1 | 100 100 |
|  |  |  | MmdD | - | -^2^ | - | - |
| propionyl-CoA 🡪 propionate | 2.8.3.27 | succinate:propionate CoA-transferase | ScpC | PRU_2762 | J4031_07180 | QVJ82124.1 | 99.6 |

^1^ Proteins analysed from the *X.* *ruminicola* 23^T^ assembly CP002006.
^2^ Determined by BLASTP searches (default settings) of MmdD subunits of Swiss-Prot proteins of *Veillonella parvula* (Q56724) and *Propionigenium modestum* (O54029).

**Table S4. Homologues of propionate pathway genes in screened Hungate1000 genomes**

| Enzymatic step | Enzyme subunit | Protein ID in 23^T 1^ | KHP1 protein homologue | AR32 (2654588202) | BP1-162 (2623620485) | Ga6B6 (2558860122) | HUN156 (2593339271) | KHT3 (2654588175) | MA2016 (2558860990) | mn60 (2623620474) | P6B4 (2558860126) | P6B11 (2558860121) | RM4 (2562617035) | TC2-24 (2623621022) | TC2-28 (2623620552) | TF2-5 (2623620458) |
| --- | --- | --- | --- | --- | --- | --- | --- | --- | --- | --- | --- | --- | --- | --- | --- | --- |
| phosphoenolpyruvate 🡪 oxaloacetate | E4.1.1.49 | PRU_2279 | QVJ79957 | Ga0104419_0693 | Ga0070636_101596 | T500DRAFT_00288 | IE21DRAFT_1952 | Ga0104360_10623 | T360DRAFT_1999 | Ga0066892_10441 | T491DRAFT_01183 | T496DRAFT_01169 | T499DRAFT_1906 | Ga0066887_0900 | Ga0066886_11711 | Ga0066888_101352 |
| oxaloacetate 🡪 malate | Mdh | PRU_2158 | QVJ80061 | Ga0104419_2508 | Ga0070636_104175 | T500DRAFT_00604 | IE21DRAFT_1368 | Ga0104360_10852 | T360DRAFT_1512 | Ga0066892_11449 | T491DRAFT_00414 | T496DRAFT_00360 | T499DRAFT_0282 | Ga0066887_1669 | Ga0066886_10943 | Ga0066888_1168 |
| malate 🡪 fumarate | E4.2.1.2A | PRU_0119 | QVJ81858 | Ga0104419_1849 | Ga0070636_10516 | T500DRAFT_02151 | IE21DRAFT_1205 | Ga0104360_10121 | T360DRAFT_2800 | Ga0066892_10391 | T491DRAFT_00620 | T496DRAFT_00621 | T499DRAFT_2197 | Ga0066887_2209 | Ga0066886_10519 | Ga0066888_107137 |
| fumarate 🡪 succinate | SdhA  SdhB  SdhC | PRU_2432  PRU_2431  PRU_2433 | QVJ79830  QVJ79831  QVJ79829 | Ga0104419_0867  Ga0104419_0866  Ga0104419_0868 | Ga0070636_101446  Ga0070636_101447  Ga0070636_101445 | T500DRAFT_00138  T500DRAFT_00139  T500DRAFT_00137 | IE21DRAFT_0310  IE21DRAFT_0309  IE21DRAFT_0311 | Ga0104360_12138  Ga0104360_12137  Ga0104360_12139 | T360DRAFT_2169  T360DRAFT_2168  T360DRAFT_2170 | Ga0066892_10697  Ga0066892_10696  Ga0066892_10698 | T491DRAFT_00766  T491DRAFT_00767  T491DRAFT_00765 | T496DRAFT_00317  T496DRAFT_00318  T496DRAFT_00316 | T499DRAFT_1762  T499DRAFT_1763  T499DRAFT_1761 | Ga0066887_1084  Ga0066887_1083  Ga0066887_1085 | Ga0066886_106144  Ga0066886_106143  Ga0066886_106145 | Ga0066888_11425  Ga0066888_11424  Ga0066888_11426 |
| succinate 🡪 succinyl- CoA | ScpC | PRU_2762 | QVJ82124 | Ga0104419_2780 | Ga0070636_101113 | T500DRAFT_00547 | IE21DRAFT_0489 | Ga0104360_10228 | T360DRAFT_1051 | Ga0066892_11628 | T491DRAFT_00325 | T496DRAFT_02472 | T499DRAFT_1511 | Ga0066887_2311 | Ga0066886_10648 | Ga0066888_105197 |
| succinyl-CoA 🡪 *R*-methylmalonyl-CoA | MutA | PRU_1640 | QVJ80503 | Ga0104419_1460 | Ga0070636_10341 | T500DRAFT_00989 | IE21DRAFT_0747 | Ga0104360_11421 | T360DRAFT_1688 | Ga0066892_101382 | T491DRAFT_01235 | T496DRAFT_01539 | T499DRAFT_0809 | Ga0066887_0739 | Ga0066886_1219 | Ga0066888_10294 |
|  | MutB | PRU_1639 | QVJ80504 | Ga0104419_1459 | Ga0070636_10342 | T500DRAFT_00990 | IE21DRAFT_0748 | Ga0104360_11422 | T360DRAFT_1687 | Ga0066892_101381 | T491DRAFT_01236 | T496DRAFT_01540 | T499DRAFT_0810 | Ga0066887_0738 | Ga0066886_1218 | Ga0066888_10295 |
| *R*-methylmalonyl-CoA 🡪 *S*-methylmalonyl-CoA | MCEE | PRU_1230 | QVJ80881 | Ga0104419_2022 | Ga0070636_102384 | T500DRAFT_02603 | IE21DRAFT_1895 | Ga0104360_12013 | T360DRAFT_0627 | Ga0066892_102243 | T491DRAFT_01782 | T496DRAFT_01962 | T499DRAFT_1205 | Ga0066887_0461 | Ga0066886_103197 | Ga0066888_10943 |
| *S*-methylmalonyl-CoA 🡪 propionyl-CoA | MmdA | PRU_1231 | QVJ80880 | Ga0104419_2021 | Ga0070636_102385 | T500DRAFT_02602 | IE21DRAFT_1896 | Ga0104360_12014 | T360DRAFT_0628 | Ga0066892_102244 | T491DRAFT_01781 | T496DRAFT_01963 | T499DRAFT_1204 | Ga0066887_0462 | Ga0066886_103198 | Ga0066888_10942 |
|  | MmdB | PRU_1644 | QVJ80499 | Ga0104419_1464 | Ga0070636_10337 | T500DRAFT_00985 | IE21DRAFT_0743 | Ga0104360_11417 | T360DRAFT_1692 | Ga0066892_101386 | T491DRAFT_01231 | T496DRAFT_02521 | T499DRAFT_0805 | Ga0066887_0743 | Ga0066886_12113 | Ga0066888_10290 |
|  | MmdC | PRU_1232  PRU_1643 | QVJ80878  QVJ80500 | Ga0104419_2019  Ga0104419_1463 | Ga0070636_102387  Ga0070636_10338 | T500DRAFT_02600  T500DRAFT_00986 | IE21DRAFT_1898  IE21DRAFT_0744 | Ga0104360_12016  Ga0104360_11418 | T360DRAFT_0630  T360DRAFT_1691 | Ga0066892_102246  Ga0066892_101385 | T491DRAFT_01779  T491DRAFT_01232 | T496DRAFT_01965  T496DRAFT_02520 | T499DRAFT_1202  T499DRAFT_0806 | Ga0066887_0464  Ga0066887_0742 | Ga0066886_103200  Ga0066886_12112 | Ga0066888_10940  Ga0066888_10291 |
|  | MmdD | - | - | - | - | - | - | - | - | - | - | - | - | - | - | - |
| propionyl-CoA 🡪 propionate | ScpC | PRU_2762 | QVJ82124 | Ga0104419_2780 | Ga0070636_101113 | T500DRAFT_00547 | IE21DRAFT_0489 | Ga0104360_10228 | T360DRAFT_1051 | Ga0066892_11628 | T491DRAFT_00325 | T496DRAFT_02472 | T499DRAFT_1511 | Ga0066887_2311 | Ga0066886_10648 | Ga0066888_105197 |

^1^ Proteins analysed from the *X.* *ruminicola* 23^T^ assembly CP002006.
^2^ Determined by BLASTP searches (default settings) of MmdD subunits of Swiss-Prot proteins of *Veillonella parvula* (Q56724) and *Propionigenium modestum* (O54029).

**Table S5. 'Cobalamin' family riboswitches predicted in the KHP1 genome**

| **Genome coordinates** | **Predicted riboswitch class** | **pHMM^1^ score** | **E-value** | **Sequence (5’-3’)** |
| --- | --- | --- | --- | --- |
| 291423..291608 (+) | cobalamin | 50.5 | 1.3e^-17^ | CAAAATAAGGTCATCTGGTGGCCGATGCCACTACGATGAAAAGGGAATACGGTGAGAATCCGTAACTGTACCTGCAGCTGTAATCCTCGCAAAAAGGGTTTGCCTGTATAACGCCACTGAGCCACAGGCTCGGGAAGGTAAGGCAGACTGAGGAAAGTCAGAAGACCTGCCGAATGTCAATTGAAG |
| 1604149-1604333 (-) | cobalamin | 30.5 | 1.9e^-11^ | TTTAGCAAATACCGAAAAAGGCTGGTCTTCAGACTTTCCTCCACTCCCAAGCGCCTTCTCACCCAAATGGGCAATGGCATTGATGCTTAGGAGCCATAATGGAGTTCACTGCTGCGGGACAGTCGGAGATTCTCACTCACATTCCCAATTAATCGCGGCTAAGCGAACCTTTACGGGCGAAGGTC |
| 2049143..2049361 (-) | cobalamin | 32.2 | 5.6e^-12^ | AAGCGGCTAAAGTTTCATCGGCAGGTCTTCTGACTTATCGTCTGGCAGCAAACGTCTTCCCAAAAATCTCAGTGACATACATGTTTGCGCCTCATAAAGGACGACTTACAGCAGCAGGAAAGGCCACGGGTAGGTGCCTTTCGGCGGGATTCTGTTCAGGATTCTCACCCGATTCCCTATTAATCTCCACATCAGGAGAACCGATTACAGCTGCAAAGA |
| 2792551..2792735 (+) | cobalamin | 17.1 | 2.4e^-07^ | GTAATATCACCCGCGAAGGTGCACGTAACTGTGCTTAATTGGGAATGTGAGTGAGAATCTCCGACTGTCCCGCAGCAGTGAACTCCATTATGGCTGTCCGACTATAGGCCATTGCCCCGTTTTGGGCGAGAAGGCGTCGGATAGTGGAGGAAAGTCTGAAGACCAGCCTTCTGCGATTTTGTTGC |

^1^ Profile hidden Markov model.

**Supplementary Table S6. Differentially expressed KHP1 genes between cobalamin treatments**

| Gene ID^1^ | BaseMean^2^ | Log_2_ fold change (+B_12_/-B_12_) | Log_2_ fold change standard error | Q-value^3^ | Gene annotation |
| --- | --- | --- | --- | --- | --- |
| J4031_01230 | 5676.6 | -6.22 | 0.23 | 1.58E-155 | sirohydrochlorin cobaltochelatase |
| J4031_01240 | 918.6 | -5.87 | 0.20 | 3.67E-194 | precorrin-3B C(17)-methyltransferase |
| J4031_01245 | 356.9 | -5.72 | 0.25 | 2.33E-116 | cobyric acid synthase |
| J4031_01235 | 1167.2 | -5.58 | 0.22 | 1.66E-143 | TonB-dependent receptor |
| J4031_01250 | 233.6 | -5.58 | 0.25 | 1.56E-104 | cobalamin biosynthesis protein CobD |
| J4031_07030 | 2409.7 | -5.22 | 0.28 | 2.82E-75 | O-acetylhomoserine aminocarboxypropyltransferase/cysteine synthase |
| J4031_01225 | 3355.9 | -4.64 | 0.16 | 6.15E-189 | TonB-dependent receptor |
| J4031_06995 | 1502.0 | -4.22 | 0.15 | 1.47E-180 | hypothetical protein cmsearch. |
| J4031_11665 | 8541.7 | -3.75 | 0.20 | 2.13E-78 | Lrp/AsnC ligand binding domain-containing protein |
| J4031_06475 | 3.0 | -3.12 | 0.90 | 2.45E-03 | tRNA-Ala |
| J4031_08510 | 348.0 | -3.11 | 0.13 | 1.21E-131 | hypothetical protein |
| J4031_08515 | 218.3 | -3.05 | 0.17 | 4.82E-74 | YncE family protein |
| J4031_08500 | 396.7 | -2.59 | 0.15 | 2.62E-63 | ABC transporter substrate-binding protein |
| J4031_08505 | 2664.4 | -2.56 | 0.19 | 1.33E-39 | DUF4465 domain-containing protein |
| J4031_08520 | 757.9 | -2.55 | 0.11 | 1.16E-119 | TonB-dependent receptor plug domain-containing protein |
| J4031_03120 | 102.1 | -2.03 | 0.25 | 8.00E-14 | hypothetical protein |
| J4031_07910 | 156.9 | -1.86 | 0.27 | 1.56E-10 | hypothetical protein |
| J4031_06940 | 260.0 | -1.53 | 0.22 | 3.07E-10 | response regulator transcription factor |
| J4031_02045 | 7.5 | -1.47 | 0.51 | 1.25E-02 | tRNA-Lys |
| J4031_07025 | 1295.0 | -1.45 | 0.19 | 1.02E-12 | cysteine synthase A |
| J4031_12275 | 90.8 | -1.38 | 0.35 | 4.80E-04 | AraC family transcriptional regulator |
| J4031_00215 | 4197.4 | -1.37 | 0.35 | 6.63E-04 | formate C-acetyltransferase |
| J4031_02090 | 138.2 | -1.36 | 0.31 | 1.17E-04 | hypothetical protein |
| J4031_01915 | 655.8 | -1.31 | 0.33 | 5.47E-04 | tRNA-Leu |
| J4031_13005 | 1521.8 | -1.27 | 0.21 | 4.48E-08 | hypothetical protein |
| J4031_05470 | 1426.6 | -1.25 | 0.38 | 4.42E-03 | RagB/SusD family nutrient uptake outer membrane protein |
| J4031_00405 | 31432.7 | -1.23 | 0.23 | 1.71E-06 | 30S ribosomal protein S1 |
| J4031_03995 | 367.6 | -1.23 | 0.19 | 9.43E-09 | AzlC family ABC transporter permease |
| J4031_00950 | 1171.0 | -1.21 | 0.20 | 4.82E-08 | 4Fe-4S binding protein |
| J4031_09205 | 1155.5 | -1.21 | 0.49 | 3.05E-02 | class I mannose-6-phosphate isomerase |
| J4031_00790 | 308.1 | -1.20 | 0.24 | 7.18E-06 | YccF domain-containing protein |
| J4031_01265 | 3841.8 | -1.20 | 0.15 | 6.35E-13 | biosynthetic-type acetolactate synthase large subunit |
| J4031_10975 | 72.4 | -1.19 | 0.43 | 1.55E-02 | hypothetical protein |
| J4031_07795 | 8942.6 | -1.19 | 0.24 | 1.03E-05 | 50S ribosomal protein L13 |
| J4031_07775 | 338.5 | -1.19 | 0.23 | 7.36E-06 | carbohydrate kinase |
| J4031_03375 | 5580.1 | -1.18 | 0.26 | 6.62E-05 | 50S ribosomal protein L9 |
| J4031_03270 | 11.9 | -1.18 | 0.48 | 3.06E-02 | tRNA-Ala |
| J4031_01260 | 4866.1 | -1.18 | 0.15 | 2.12E-13 | dihydroxy-acid dehydratase |
| J4031_01920 | 8037.6 | -1.18 | 0.30 | 5.92E-04 | tRNA-Leu |
| J4031_03990 | 46.1 | -1.18 | 0.29 | 3.94E-04 | AzlD domain-containing protein |
| J4031_06455 | 11111.2 | -1.18 | 0.23 | 4.26E-06 | DUF177 domain-containing protein |
| J4031_03275 | 54.8 | -1.17 | 0.32 | 1.37E-03 | tRNA-Ile |
| J4031_10215 | 1954.5 | -1.16 | 0.26 | 9.89E-05 | tRNA-Thr |
| J4031_11015 | 59.6 | -1.16 | 0.21 | 6.54E-07 | diaminopimelate epimerase |
| J4031_08200 | 532.4 | -1.16 | 0.22 | 1.53E-06 | TonB-dependent receptor |
| J4031_10350 | 47.7 | -1.15 | 0.37 | 6.99E-03 | helix-turn-helix transcriptional regulator |
| J4031_07780 | 10801.8 | -1.15 | 0.24 | 1.92E-05 | elongation factor Ts |
| J4031_10250 | 7457.5 | -1.14 | 0.26 | 1.25E-04 | 50S ribosomal protein L10 |
| J4031_10460 | 2656.4 | -1.13 | 0.25 | 6.43E-05 | 30S ribosomal protein S5 |
| J4031_07785 | 7551.2 | -1.13 | 0.26 | 1.25E-04 | 30S ribosomal protein S2 |
| J4031_03280 | 304.0 | -1.13 | 0.41 | 1.76E-02 | 16S ribosomal RNA |
| J4031_11010 | 193.9 | -1.13 | 0.24 | 3.04E-05 | glutamate synthase subunit beta |
| J4031_10285 | 11862.6 | -1.13 | 0.28 | 4.92E-04 | 30S ribosomal protein S10 |
| J4031_05660 | 30087.5 | -1.12 | 0.29 | 5.83E-04 | 30S ribosomal protein S15 |
| J4031_05050 | 19146.1 | -1.12 | 0.23 | 1.71E-05 | 50S ribosomal protein L25/general stress protein |
| J4031_05045 | 675.1 | -1.11 | 0.30 | 1.08E-03 | aminoacyl-tRNA hydrolase |
| J4031_03330 | 1890.5 | -1.10 | 0.36 | 8.41E-03 | tRNA methylthiotransferase MtaB |
| J4031_00015 | 1682.6 | -1.09 | 0.33 | 4.37E-03 | TonB-dependent receptor |
| J4031_07790 | 3114.2 | -1.09 | 0.26 | 2.56E-04 | 30S ribosomal protein S9 |
| J4031_10470 | 3032.8 | -1.09 | 0.26 | 2.22E-04 | 50S ribosomal protein L15 |
| J4031_12485 | 3138.1 | -1.08 | 0.39 | 1.52E-02 | sulfate permease |
| J4031_06240 | 352.4 | -1.08 | 0.29 | 9.83E-04 | MATE family efflux transporter |
| J4031_02580 | 771.6 | -1.08 | 0.30 | 1.56E-03 | tRNA-Glu |
| J4031_10970 | 148.5 | -1.08 | 0.39 | 1.70E-02 | asparagine synthase B |
| J4031_11005 | 853.2 | -1.08 | 0.22 | 1.70E-05 | glutamate synthase large subunit |
| J4031_10015 | 11.4 | -1.06 | 0.40 | 2.23E-02 | tRNA-Ile |
| J4031_03400 | 4826.2 | -1.06 | 0.23 | 5.80E-05 | HAMP domain-containing histidine kinase |
| J4031_00945 | 261.0 | -1.06 | 0.22 | 2.61E-05 | tetratricopeptide repeat protein |
| J4031_08205 | 498.7 | -1.05 | 0.20 | 2.93E-06 | TonB-dependent receptor |
| J4031_03460 | 3720.0 | -1.05 | 0.29 | 1.51E-03 | ABC-F family ATP-binding cassette domain-containing protein |
| J4031_03325 | 924.8 | -1.05 | 0.36 | 1.04E-02 | glycosyltransferase family 2 protein |
| J4031_10240 | 16316.2 | -1.05 | 0.23 | 7.58E-05 | 50S ribosomal protein L11 |
| J4031_10255 | 22134.3 | -1.03 | 0.28 | 1.51E-03 | 50S ribosomal protein L7/L12 |
| J4031_13115 | 7848.1 | -1.03 | 0.30 | 2.77E-03 | 30S ribosomal protein S16 |
| J4031_05745 | 22.7 | -1.02 | 0.41 | 2.93E-02 | hypothetical protein |
| J4031_07150 | 2284.1 | -1.02 | 0.39 | 2.46E-02 | pyridoxal 5'-phosphate synthase glutaminase subunit PdxT |
| J4031_04815 | 3182.6 | -1.01 | 0.21 | 2.22E-05 | Smr/MutS family protein |
| J4031_00210 | 377.6 | -1.01 | 0.36 | 1.45E-02 | pyruvate formate lyase-activating protein |
| J4031_09280 | 8492.7 | -1.01 | 0.22 | 6.62E-05 | 30S ribosomal protein S20 |
| J4031_10245 | 13752.4 | -1.01 | 0.25 | 4.35E-04 | 50S ribosomal protein L1 |
| J4031_11560 | 8446.3 | -1.01 | 0.33 | 8.36E-03 | efflux RND transporter permease subunit |
| J4031_04375 | 530.8 | -1.00 | 0.21 | 1.94E-05 | sulfur carrier protein ThiS analysis using gene prediction method: cmsearch. |
| J4031_03195 | 2292.4 | -1.00 | 0.19 | 4.78E-06 | putative transporter |
| J4031_07990 | 804.9 | 1.00 | 0.32 | 6.56E-03 | ABC transporter substrate-binding protein |
| J4031_04330 | 766.0 | 1.01 | 0.34 | 9.28E-03 | carboxylesterase family protein |
| J4031_12660 | 2234.5 | 1.01 | 0.40 | 2.96E-02 | D-xylose transporter XylE |
| J4031_11835 | 762.9 | 1.01 | 0.22 | 4.46E-05 | glycosyltransferase family 2 protein |
| J4031_12475 | 1016.0 | 1.01 | 0.34 | 9.11E-03 | exodeoxyribonuclease III |
| J4031_14120 | 71.0 | 1.02 | 0.26 | 6.09E-04 | nucleotidyltransferase family protein |
| J4031_03980 | 701.8 | 1.02 | 0.22 | 4.63E-05 | hypothetical protein |
| J4031_12470 | 636.8 | 1.02 | 0.30 | 3.38E-03 | hypothetical protein |
| J4031_00440 | 629.1 | 1.02 | 0.38 | 2.05E-02 | restriction endonuclease subunit S |
| J4031_11125 | 108.1 | 1.02 | 0.38 | 1.96E-02 | hypothetical protein |
| J4031_12125 | 2052.1 | 1.02 | 0.24 | 1.85E-04 | hypothetical protein |
| J4031_10080 | 931.6 | 1.03 | 0.37 | 1.64E-02 | DUF5110 domain-containing protein |
| J4031_03595 | 131.4 | 1.03 | 0.32 | 4.37E-03 | AmmeMemoRadiSam system radical SAM enzyme |
| J4031_04710 | 458.3 | 1.03 | 0.39 | 2.03E-02 | GNAT family N-acetyltransferase |
| J4031_02285 | 1119.1 | 1.03 | 0.40 | 2.66E-02 | type II restriction endonuclease subunit M |
| J4031_10365 | 89.7 | 1.03 | 0.34 | 7.80E-03 | DNA-binding protein |
| J4031_07480 | 110.6 | 1.03 | 0.44 | 4.23E-02 | family 43 glycosylhydrolase |
| J4031_12315 | 3983.3 | 1.03 | 0.36 | 1.33E-02 | ROK family protein |
| J4031_06610 | 120.8 | 1.04 | 0.31 | 3.99E-03 | DUF2029 domain-containing protein |
| J4031_05465 | 287.4 | 1.04 | 0.41 | 2.76E-02 | NAD(P)/FAD-dependent oxidoreductase |
| J4031_00450 | 1474.5 | 1.04 | 0.28 | 1.29E-03 | type I restriction endonuclease subunit R |
| J4031_13305 | 1370.7 | 1.04 | 0.36 | 1.13E-02 | hypothetical protein |
| J4031_12575 | 694.2 | 1.04 | 0.42 | 3.31E-02 | hypothetical protein |
| J4031_10195 | 11832.6 | 1.04 | 0.39 | 1.91E-02 | ribosome-associated translation inhibitor RaiA |
| J4031_03150 | 622.4 | 1.04 | 0.44 | 3.99E-02 | Z1 domain-containing protein |
| J4031_07960 | 1151.2 | 1.04 | 0.29 | 1.62E-03 | hypothetical protein |
| J4031_09245 | 962.1 | 1.04 | 0.39 | 1.97E-02 | phosphate butyryltransferase |
| J4031_10950 | 25.5 | 1.04 | 0.39 | 2.07E-02 | DUF3836 domain-containing protein |
| J4031_13320 | 1346.5 | 1.05 | 0.22 | 3.16E-05 | hypothetical protein |
| J4031_11910 | 57.3 | 1.05 | 0.30 | 2.33E-03 | N-acetylmuramoyl-L-alanine amidase |
| J4031_08545 | 1593.7 | 1.05 | 0.24 | 1.51E-04 | aldo/keto reductase |
| J4031_11930 | 53.6 | 1.05 | 0.26 | 4.32E-04 | AAA family ATPase |
| J4031_00465 | 125.8 | 1.05 | 0.46 | 4.85E-02 | hypothetical protein |
| J4031_10075 | 334.4 | 1.05 | 0.36 | 1.13E-02 | SusF/SusE family outer membrane protein |
| J4031_06050 | 1143.3 | 1.05 | 0.32 | 4.52E-03 | TonB-dependent receptor plug domain-containing protein |
| J4031_11855 | 282.1 | 1.05 | 0.21 | 8.53E-06 | 2-C-methyl-D-erythritol 4-phosphate cytidylyltransferase |
| J4031_13720 | 2520.5 | 1.06 | 0.13 | 3.35E-14 | DUF4842 domain-containing protein |
| J4031_11950 | 819.0 | 1.06 | 0.17 | 8.10E-09 | molecular chaperone Tir |
| J4031_04440 | 76.9 | 1.06 | 0.38 | 1.60E-02 | leucine-rich repeat protein |
| J4031_04390 | 571.4 | 1.06 | 0.28 | 1.05E-03 | histidine acid phosphatase |
| J4031_05680 | 369.5 | 1.06 | 0.28 | 8.83E-04 | M6 family metalloprotease domain-containing protein |
| J4031_00260 | 2628.8 | 1.06 | 0.16 | 1.54E-09 | type II CRISPR RNA-guided endonuclease Cas9 |
| J4031_13285 | 2587.1 | 1.06 | 0.42 | 2.73E-02 | DNA-binding protein |
| J4031_10065 | 1905.2 | 1.06 | 0.30 | 2.25E-03 | hypothetical protein |
| J4031_10725 | 2367.2 | 1.07 | 0.28 | 6.75E-04 | glycine cleavage system aminomethyltransferase |
| J4031_09895 | 273.6 | 1.07 | 0.29 | 1.42E-03 | hypothetical protein |
| J4031_11130 | 122.3 | 1.07 | 0.39 | 1.64E-02 | hypothetical protein |
| J4031_09785 | 71.2 | 1.07 | 0.32 | 3.98E-03 | hypothetical protein |
| J4031_12100 | 1728.7 | 1.07 | 0.18 | 1.45E-07 | hypothetical protein |
| J4031_08005 | 1403.2 | 1.07 | 0.39 | 1.62E-02 | right-handed parallel beta-helix repeat-containing protein |
| J4031_13710 | 294.3 | 1.07 | 0.26 | 3.21E-04 | hypothetical protein |
| J4031_10730 | 990.6 | 1.07 | 0.25 | 1.74E-04 | glycine cleavage system protein GcvH |
| J4031_10945 | 71.4 | 1.08 | 0.39 | 1.63E-02 | FtsX-like permease family protein |
| J4031_12770 | 661.5 | 1.08 | 0.32 | 3.32E-03 | endonuclease/exonuclease/phosphatase family protein |
| J4031_13325 | 70.0 | 1.08 | 0.26 | 2.72E-04 | hypothetical protein |
| J4031_05485 | 24.8 | 1.08 | 0.45 | 3.67E-02 | hypothetical protein |
| J4031_02545 | 1304.6 | 1.08 | 0.20 | 9.49E-07 | AAA family ATPase |
| J4031_12390 | 4138.3 | 1.08 | 0.37 | 1.01E-02 | large-conductance mechanosensitive channel protein MscL |
| J4031_11965 | 335.5 | 1.09 | 0.17 | 2.50E-09 | hypothetical protein |
| J4031_11515 | 277.5 | 1.09 | 0.26 | 2.47E-04 | hypothetical protein |
| J4031_13605 | 795.2 | 1.10 | 0.26 | 2.45E-04 | DUF4422 domain-containing protein |
| J4031_10525 | 1012.8 | 1.10 | 0.39 | 1.33E-02 | septal ring lytic transglycosylase RlpA family protein |
| J4031_09885 | 549.0 | 1.10 | 0.29 | 6.63E-04 | PD40 domain-containing protein |
| J4031_09850 | 414.2 | 1.11 | 0.31 | 1.76E-03 | alpha-xylosidase |
| J4031_12075 | 689.9 | 1.11 | 0.21 | 3.93E-06 | hypothetical protein |
| J4031_12090 | 970.7 | 1.11 | 0.22 | 1.23E-05 | MBL fold metallo-hydrolase |
| J4031_08220 | 1052.2 | 1.11 | 0.26 | 1.38E-04 | transporter substrate-binding domain-containing protein |
| J4031_03580 | 128.4 | 1.12 | 0.31 | 1.45E-03 | DUF3874 domain-containing protein |
| J4031_03535 | 2001.8 | 1.12 | 0.27 | 3.21E-04 | phage holin family protein |
| J4031_11955 | 995.9 | 1.12 | 0.17 | 1.11E-09 | hypothetical protein |
| J4031_00460 | 173.4 | 1.12 | 0.27 | 2.18E-04 | hypothetical protein |
| J4031_10690 | 561.7 | 1.12 | 0.23 | 1.96E-05 | hypothetical protein |
| J4031_12005 | 1069.1 | 1.12 | 0.12 | 3.81E-19 | polysaccharide pyruvyl transferase family protein |
| J4031_12000 | 1441.1 | 1.12 | 0.14 | 2.35E-14 | Coenzyme F420 hydrogenase/dehydrogenase, beta subunit C-terminal domain |
| J4031_08085 | 1716.7 | 1.13 | 0.37 | 8.69E-03 | GH92 family glycosyl hydrolase |
| J4031_09770 | 217.4 | 1.13 | 0.27 | 2.35E-04 | peptidase C13 |
| J4031_11960 | 1025.7 | 1.13 | 0.13 | 5.25E-16 | hypothetical protein |
| J4031_09910 | 339.2 | 1.13 | 0.28 | 3.79E-04 | hypothetical protein |
| J4031_12045 | 603.0 | 1.13 | 0.13 | 5.97E-16 | O-antigen ligase family protein |
| J4031_08970 | 422.6 | 1.14 | 0.42 | 1.87E-02 | thioredoxin-dependent thiol peroxidase |
| J4031_10085 | 1323.2 | 1.14 | 0.40 | 1.40E-02 | discoidin domain-containing protein |
| J4031_13595 | 699.1 | 1.14 | 0.25 | 6.00E-05 | glycosyltransferase family 4 protein |
| J4031_07405 | 13.0 | 1.14 | 0.43 | 1.91E-02 | glycosyl hydrolase family 76 |
| J4031_07640 | 635.9 | 1.15 | 0.43 | 2.11E-02 | helix-turn-helix domain-containing protein |
| J4031_13755 | 749.5 | 1.15 | 0.24 | 2.43E-05 | glycosyltransferase |
| J4031_09780 | 39.1 | 1.15 | 0.28 | 2.60E-04 | hypothetical protein |
| J4031_07230 | 1856.6 | 1.15 | 0.21 | 1.29E-06 | transporter substrate-binding domain-containing protein |
| J4031_04450 | 92.1 | 1.16 | 0.42 | 1.75E-02 | hypothetical protein |
| J4031_06855 | 620.6 | 1.16 | 0.40 | 1.25E-02 | aldo/keto reductase |
| J4031_11075 | 1563.2 | 1.16 | 0.37 | 6.19E-03 | DUF853 family protein |
| J4031_02280 | 37.5 | 1.16 | 0.37 | 6.97E-03 | DUF262 domain-containing protein analysis using gene prediction method: Protein Homology. |
| J4031_11820 | 1829.3 | 1.16 | 0.16 | 1.86E-11 | polysaccharide pyruvyl transferase family protein |
| J4031_12030 | 694.3 | 1.16 | 0.12 | 5.96E-19 | PIG-L family deacetylase |
| J4031_07600 | 901.4 | 1.16 | 0.42 | 1.53E-02 | glycosyl hydrolase 53 family protein |
| J4031_13725 | 2161.4 | 1.16 | 0.12 | 1.57E-19 | DUF4842 domain-containing protein |
| J4031_01375 | 283.6 | 1.16 | 0.33 | 2.09E-03 | TIGR02172 family protein |
| J4031_13315 | 316.0 | 1.16 | 0.26 | 1.10E-04 | phage antirepressor KilAC domain-containing protein |
| J4031_13310 | 370.2 | 1.17 | 0.24 | 1.16E-05 | Bro-N domain-containing protein |
| J4031_09625 | 2356.6 | 1.17 | 0.25 | 2.69E-05 | substrate-binding domain-containing protein |
| J4031_10955 | 70.3 | 1.17 | 0.31 | 1.09E-03 | FtsX-like permease family protein |
| J4031_12460 | 2413.7 | 1.17 | 0.36 | 4.25E-03 | glycosyltransferase family 4 protein |
| J4031_11425 | 1421.2 | 1.18 | 0.28 | 2.40E-04 | DNA-binding protein |
| J4031_07315 | 7.1 | 1.18 | 0.50 | 4.17E-02 | SusC/RagA family TonB-linked outer membrane protein |
| J4031_11990 | 917.8 | 1.18 | 0.20 | 2.21E-07 | polysaccharide biosynthesis protein |
| J4031_00860 | 905.3 | 1.19 | 0.51 | 4.49E-02 | GH92 family glycosyl hydrolase |
| J4031_12320 | 2824.9 | 1.19 | 0.41 | 1.11E-02 | ROK family protein |
| J4031_02315 | 809.8 | 1.19 | 0.20 | 1.27E-07 | DUF488 domain-containing protein |
| J4031_06860 | 275.4 | 1.19 | 0.38 | 5.90E-03 | amidohydrolase |
| J4031_04615 | 352.9 | 1.19 | 0.35 | 2.98E-03 | tetratricopeptide repeat protein |
| J4031_12465 | 4574.6 | 1.19 | 0.32 | 1.23E-03 | glycoside hydrolase family 57 protein |
| J4031_02540 | 482.4 | 1.20 | 0.14 | 1.60E-14 | hypothetical protein |
| J4031_12570 | 732.2 | 1.20 | 0.18 | 1.49E-09 | FAD-dependent monooxygenase |
| J4031_11830 | 743.7 | 1.20 | 0.20 | 4.05E-08 | glycosyltransferase family 2 protein |
| J4031_08550 | 1466.5 | 1.20 | 0.22 | 1.12E-06 | 4Fe-4S binding protein |
| J4031_12845 | 86.1 | 1.20 | 0.52 | 4.41E-02 | RagB/SusD family nutrient uptake outer membrane protein |
| J4031_02310 | 617.6 | 1.21 | 0.17 | 3.09E-11 | hypothetical protein |
| J4031_12455 | 6478.7 | 1.21 | 0.34 | 1.84E-03 | glycogen debranching enzyme family protein |
| J4031_10070 | 1395.0 | 1.21 | 0.39 | 7.41E-03 | DUF5110 domain-containing protein |
| J4031_10740 | 2438.1 | 1.21 | 0.37 | 4.16E-03 | aminomethyl-transferring glycine dehydrogenase subunit GcvPB |
| J4031_11735 | 1309.7 | 1.21 | 0.39 | 7.41E-03 | HAMP domain-containing histidine kinase |
| J4031_10965 | 55.8 | 1.21 | 0.39 | 7.63E-03 | ABC transporter ATP-binding protein |
| J4031_09765 | 60.7 | 1.21 | 0.23 | 3.65E-06 | hypothetical protein |
| J4031_11845 | 427.3 | 1.22 | 0.17 | 1.71E-10 | glycosyltransferase family 2 protein |
| J4031_00385 | 469.2 | 1.23 | 0.40 | 7.43E-03 | hypothetical protein |
| J4031_07430 | 28.4 | 1.23 | 0.34 | 1.78E-03 | glycoside hydrolase family 125 protein |
| J4031_01735 | 906.3 | 1.24 | 0.36 | 2.67E-03 | ABC transporter substrate-binding protein |
| J4031_07985 | 1252.0 | 1.24 | 0.30 | 2.56E-04 | SpoIIE family protein phosphatase |
| J4031_03145 | 182.8 | 1.25 | 0.42 | 1.00E-02 | PD-(D/E)XK motif protein |
| J4031_03405 | 9309.4 | 1.25 | 0.42 | 9.94E-03 | elongation factor G |
| J4031_12855 | 25.1 | 1.25 | 0.47 | 2.10E-02 | glycoside hydrolase family 30 protein |
| J4031_12990 | 2103.0 | 1.26 | 0.39 | 4.43E-03 | hypothetical protein |
| J4031_09250 | 899.6 | 1.26 | 0.45 | 1.41E-02 | butyrate kinase |
| J4031_02530 | 400.5 | 1.27 | 0.19 | 1.49E-09 | hypothetical protein |
| J4031_10595 | 8.7 | 1.27 | 0.50 | 2.77E-02 | family 43 glycosylhydrolase |
| J4031_06045 | 408.2 | 1.27 | 0.34 | 9.41E-04 | DUF4249 domain-containing protein |
| J4031_10560 | 1537.8 | 1.27 | 0.40 | 5.47E-03 | hypothetical protein |
| J4031_02265 | 21.9 | 1.27 | 0.45 | 1.39E-02 | ATP-binding protein |
| J4031_02275 | 29.9 | 1.27 | 0.45 | 1.28E-02 | DUF262 domain-containing protein |
| J4031_10700 | 298.7 | 1.27 | 0.20 | 1.46E-08 | TlpA family protein disulfide reductase |
| J4031_12290 | 221.6 | 1.28 | 0.34 | 9.45E-04 | MGMT family protein |
| J4031_12850 | 37.7 | 1.28 | 0.43 | 1.03E-02 | xylanase |
| J4031_02270 | 83.1 | 1.28 | 0.36 | 1.56E-03 | hypothetical protein |
| J4031_12840 | 169.8 | 1.28 | 0.44 | 1.08E-02 | TonB-dependent receptor |
| J4031_13280 | 132.0 | 1.28 | 0.32 | 3.61E-04 | smalltalk protein |
| J4031_10710 | 3002.7 | 1.28 | 0.47 | 1.72E-02 | MIP family channel protein |
| J4031_11920 | 16.2 | 1.29 | 0.31 | 3.13E-04 | hypothetical protein |
| J4031_13715 | 158.4 | 1.29 | 0.42 | 7.19E-03 | hypothetical protein |
| J4031_03140 | 518.1 | 1.29 | 0.40 | 5.07E-03 | AIPR family protein |
| J4031_09890 | 166.3 | 1.29 | 0.37 | 2.40E-03 | DUF4369 domain-containing protein |
| J4031_07820 | 1438.1 | 1.30 | 0.23 | 7.16E-07 | 9-O-acetylesterase |
| J4031_01775 | 99.0 | 1.30 | 0.23 | 5.20E-07 | PriCT-2 domain-containing protein |
| J4031_12010 | 427.5 | 1.30 | 0.18 | 1.43E-11 | acyltransferase |
| J4031_12070 | 1201.5 | 1.30 | 0.22 | 8.11E-08 | WecB/TagA/CpsF family glycosyltransferase |
| J4031_04460 | 128.0 | 1.31 | 0.25 | 3.69E-06 | hypothetical protein |
| J4031_12025 | 1147.7 | 1.31 | 0.16 | 3.02E-15 | glycosyltransferase family 4 protein |
| J4031_04640 | 9.6 | 1.33 | 0.53 | 2.83E-02 | hypothetical protein |
| J4031_01760 | 13.8 | 1.34 | 0.50 | 2.04E-02 | hypothetical protein |
| J4031_08055 | 302.7 | 1.35 | 0.34 | 4.28E-04 | sialate O-acetylesterase |
| J4031_11825 | 1403.5 | 1.35 | 0.21 | 4.10E-09 | glycosyltransferase family 2 protein |
| J4031_12015 | 413.1 | 1.35 | 0.17 | 3.60E-14 | serine acetyltransferase |
| J4031_04600 | 296.3 | 1.36 | 0.39 | 2.29E-03 | DUF4062 domain-containing protein |
| J4031_09255 | 738.1 | 1.36 | 0.40 | 2.80E-03 | HAMP domain-containing protein |
| J4031_12295 | 253.8 | 1.36 | 0.41 | 3.99E-03 | NAD(P)H-dependent oxidoreductase |
| J4031_02535 | 168.3 | 1.36 | 0.25 | 1.21E-06 | sigma-70 family RNA polymerase sigma factor |
| J4031_10745 | 1191.1 | 1.37 | 0.31 | 9.99E-05 | NAD(P)/FAD-dependent oxidoreductase |
| J4031_04285 | 611.0 | 1.37 | 0.56 | 3.43E-02 | beta-glucosidase BglX |
| J4031_08905 | 1945.9 | 1.37 | 0.47 | 1.14E-02 | cupin domain-containing protein |
| J4031_06875 | 681.6 | 1.38 | 0.39 | 2.29E-03 | glycoside hydrolase family 97 protein |
| J4031_12050 | 729.1 | 1.38 | 0.15 | 3.72E-18 | glycosyltransferase family 2 protein |
| J4031_06870 | 250.4 | 1.38 | 0.41 | 2.93E-03 | L-fucose:H+ symporter permease |
| J4031_07585 | 507.2 | 1.38 | 0.47 | 1.00E-02 | glycoside hydrolase 43 family protein |
| J4031_02985 | 92.6 | 1.38 | 0.43 | 4.90E-03 | beta-galactosidase |
| J4031_12565 | **5857.3** | **1.38** | **0.29** | **3.19E-05** | **methylmalonyl-CoA mutase** |
| J4031_10565 | 492.4 | 1.39 | 0.33 | 1.93E-04 | carboxylesterase/lipase family protein |
| J4031_00855 | 680.4 | 1.39 | 0.53 | 2.20E-02 | copper homeostasis protein CutC |
| J4031_13780 | 251.1 | 1.40 | 0.19 | 2.36E-11 | hypothetical protein |
| J4031_09900 | 375.3 | 1.40 | 0.25 | 8.51E-07 | PD40 domain-containing protein |
| J4031_08915 | 66.2 | 1.40 | 0.47 | 8.98E-03 | DNA-binding protein |
| J4031_04465 | 144.2 | 1.40 | 0.30 | 4.41E-05 | NADAR family protein |
| J4031_09905 | 400.2 | 1.40 | 0.29 | 2.48E-05 | PD40 domain-containing protein |
| J4031_14260 | 1033.0 | 1.40 | 0.44 | 5.45E-03 | HAMP domain-containing protein |
| J4031_12020 | 965.7 | 1.40 | 0.14 | 1.64E-20 | glycosyltransferase family 4 protein |
| J4031_12060 | 350.2 | 1.41 | 0.16 | 8.63E-16 | acyltransferase |
| J4031_06830 | 480.0 | 1.42 | 0.38 | 1.21E-03 | ABC transporter substrate-binding protein |
| J4031_04545 | 15.4 | 1.43 | 0.46 | 7.23E-03 | hypothetical protein |
| J4031_10695 | 241.3 | 1.43 | 0.21 | 5.86E-10 | thioredoxin family protein |
| J4031_05930 | 1562.5 | 1.43 | 0.28 | 8.61E-06 | transporter substrate-binding domain-containing protein |
| J4031_08480 | 1177.7 | 1.44 | 0.50 | 1.23E-02 | alpha-galactosidase |
| J4031_01360 | 1039.1 | 1.44 | 0.29 | 8.56E-06 | DUF3256 family protein |
| J4031_09260 | 792.9 | 1.44 | 0.40 | 1.65E-03 | HAMP domain-containing protein |
| J4031_05920 | 1317.8 | 1.45 | 0.29 | 8.62E-06 | PAS domain S-box protein |
| J4031_00455 | 18.8 | 1.45 | 0.48 | 9.23E-03 | hypothetical protein |
| J4031_12665 | 1582.6 | 1.45 | 0.44 | 3.73E-03 | response regulator |
| J4031_09775 | 100.2 | 1.46 | 0.24 | 4.25E-08 | hypothetical protein |
| J4031_03135 | 217.8 | 1.46 | 0.44 | 3.72E-03 | leucine-rich repeat domain-containing protein |
| J4031_05925 | 1215.7 | 1.46 | 0.27 | 2.36E-06 | PAS domain-containing sensor histidine kinase |
| J4031_05935 | 1772.8 | 1.47 | 0.34 | 1.32E-04 | transporter substrate-binding domain-containing protein |
| J4031_12140 | 106.4 | 1.47 | 0.54 | 1.73E-02 | glycoside hydrolase family 130 protein |
| J4031_07615 | 5.5 | 1.47 | 0.57 | 2.45E-02 | family 43 glycosylhydrolase |
| J4031_12740 | 720.7 | 1.48 | 0.40 | 1.08E-03 | HAMP domain-containing histidine kinase |
| J4031_08910 | 65.6 | 1.49 | 0.50 | 9.30E-03 | N-acetylmuramoyl-L-alanine amidase |
| J4031_00390 | 359.8 | 1.49 | 0.50 | 8.94E-03 | HAMP domain-containing protein |
| J4031_08835 | 12.4 | 1.49 | 0.65 | 4.85E-02 | DUF2264 domain-containing protein |
| J4031_05510 | 15.3 | 1.49 | 0.45 | 3.76E-03 | hypothetical protein |
| J4031_12325 | 3321.4 | 1.49 | 0.42 | 1.66E-03 | ROK family protein |
| J4031_08850 | 29.4 | 1.50 | 0.57 | 2.21E-02 | glycoside hydrolase family 88 protein |
| J4031_13185 | 38.2 | 1.50 | 0.42 | 1.59E-03 | flavodoxin family protein |
| J4031_12065 | 995.7 | 1.52 | 0.21 | 1.97E-11 | glycosyltransferase |
| J4031_00395 | 453.1 | 1.52 | 0.53 | 1.23E-02 | sensor histidine kinase |
| J4031_05880 | 1683.1 | 1.52 | 0.40 | 8.46E-04 | alpha-glucuronidase |
| J4031_12835 | 124.6 | 1.53 | 0.46 | 3.88E-03 | hypothetical protein |
| J4031_11035 | 22.1 | 1.53 | 0.59 | 2.37E-02 | CotH kinase family protein |
| J4031_12560 | **5293.5** | **1.54** | **0.28** | **9.11E-07** | **methylmalonyl-CoA mutase small subunit** |
| J4031_11995 | 1429.8 | 1.55 | 0.25 | 2.70E-08 | glycosyltransferase family 2 protein |
| J4031_07200 | 1810.1 | 1.55 | 0.37 | 2.18E-04 | mannose-1-phosphate guanylyltransferase |
| J4031_12760 | 3629.4 | 1.55 | 0.50 | 7.01E-03 | FAD-binding oxidoreductase |
| J4031_13180 | 112.0 | 1.56 | 0.41 | 9.92E-04 | alpha/beta hydrolase |
| J4031_09790 | 23.9 | 1.56 | 0.45 | 2.38E-03 | cyclohexadienyl dehydratase |
| J4031_10040 | 662.8 | 1.56 | 0.37 | 2.35E-04 | cob(I)yrinic acid a,c-diamide adenosyltransferase |
| J4031_01740 | 1252.2 | 1.56 | 0.39 | 4.08E-04 | SpoIIE family protein phosphatase |
| J4031_07595 | 475.3 | 1.57 | 0.44 | 1.94E-03 | acetylxylan esterase |
| J4031_13200 | 97.3 | 1.58 | 0.38 | 3.00E-04 | 4Fe-4S dicluster domain-containing protein |
| J4031_07975 | 8.5 | 1.58 | 0.59 | 1.91E-02 | P1 family peptidase |
| J4031_08095 | 1421.3 | 1.59 | 0.34 | 3.05E-05 | GH92 family glycosyl hydrolase |
| J4031_04630 | 58.6 | 1.59 | 0.40 | 4.47E-04 | TIR domain-containing protein |
| J4031_07340 | 1048.0 | 1.59 | 0.66 | 3.53E-02 | ABC transporter substrate-binding protein |
| J4031_04625 | 40.2 | 1.59 | 0.37 | 1.82E-04 | DNA/RNA non-specific endonuclease |
| J4031_04585 | 951.3 | 1.60 | 0.44 | 1.51E-03 | hypothetical protein |
| J4031_07825 | 836.1 | 1.61 | 0.23 | 6.60E-11 | NUDIX domain-containing protein |
| J4031_04605 | 709.1 | 1.61 | 0.44 | 1.51E-03 | SEL1-like repeat protein |
| J4031_04620 | 244.5 | 1.61 | 0.40 | 4.38E-04 | tetratricopeptide repeat protein |
| J4031_03585 | 97.7 | 1.61 | 0.46 | 2.23E-03 | hypothetical protein |
| J4031_14090 | 495.2 | 1.62 | 0.49 | 3.67E-03 | sulfide/dihydroorotate dehydrogenase-like |
| J4031_07425 | 13.3 | 1.63 | 0.56 | 1.07E-02 | GH92 family glycosyl hydrolase |
| J4031_12765 | 1932.9 | 1.63 | 0.41 | 5.04E-04 | carboxylesterase family protein |
| J4031_04650 | 471.2 | 1.63 | 0.54 | 8.81E-03 | hypothetical protein |
| J4031_07490 | 831.2 | 1.64 | 0.47 | 2.39E-03 | response regulator |
| J4031_04305 | 6.3 | 1.64 | 0.67 | 3.41E-02 | anaerobic sulfatase maturase |
| J4031_04580 | 837.2 | 1.65 | 0.43 | 7.61E-04 | hypothetical protein |
| J4031_08060 | 413.0 | 1.65 | 0.51 | 5.07E-03 | MFS transporter |
| J4031_01105 | 998.4 | 1.65 | 0.44 | 9.81E-04 | SusC/RagA family TonB-linked outer membrane protein |
| J4031_08755 | 5180.2 | 1.65 | 0.41 | 3.42E-04 | TonB-dependent receptor |
| J4031_04610 | 285.0 | 1.66 | 0.39 | 1.75E-04 | DUF4062 domain-containing protein |
| J4031_04635 | 126.5 | 1.67 | 0.34 | 1.70E-05 | hypothetical protein |
| J4031_08090 | 694.7 | 1.68 | 0.32 | 4.18E-06 | HAMP domain-containing histidine kinase |
| J4031_07140 | 299.8 | 1.70 | 0.49 | 2.33E-03 | DUF4421 family protein |
| J4031_07145 | 488.2 | 1.70 | 0.44 | 6.98E-04 | DUF4982 domain-containing protein |
| J4031_05490 | 83.8 | 1.71 | 0.31 | 1.30E-06 | hypothetical protein |
| J4031_08050 | 261.3 | 1.72 | 0.42 | 2.77E-04 | MFS transporter |
| J4031_06825 | 1135.2 | 1.72 | 0.37 | 4.02E-05 | SpoIIE family protein phosphatase |
| J4031_12055 | 507.5 | 1.73 | 0.28 | 2.53E-08 | glycosyl transferase |
| J4031_07995 | 5100.5 | 1.73 | 0.57 | 7.65E-03 | RagB/SusD family nutrient uptake outer membrane protein |
| J4031_11430 | 66.2 | 1.73 | 0.32 | 1.99E-06 | smalltalk protein |
| J4031_14095 | 594.1 | 1.73 | 0.51 | 2.70E-03 | FAD-dependent oxidoreductase |
| J4031_07330 | 796.2 | 1.74 | 0.52 | 3.83E-03 | glycosyl hydrolase 115 family protein |
| J4031_12780 | 1889.0 | 1.74 | 0.55 | 6.09E-03 | RagB/SusD family nutrient uptake outer membrane protein |
| J4031_01580 | 7.6 | 1.74 | 0.75 | 4.52E-02 | hypothetical protein |
| J4031_11690 | 937.4 | 1.75 | 0.49 | 1.88E-03 | phosphatase PAP2 family protein |
| J4031_08070 | 62.5 | 1.75 | 0.59 | 1.02E-02 | sigma-70 family RNA polymerase sigma factor |
| J4031_07720 | 316.7 | 1.76 | 0.68 | 2.42E-02 | glycoside hydrolase family 3 C-terminal domain-containing protein |
| J4031_07620 | 17.4 | 1.77 | 0.67 | 2.10E-02 | glycoside hydrolase family 43 protein |
| J4031_07980 | 1624.9 | 1.77 | 0.31 | 3.60E-07 | histidine-type phosphatase |
| J4031_07370 | 34.5 | 1.78 | 0.51 | 2.48E-03 | hypothetical protein |
| J4031_08900 | 885.3 | 1.78 | 0.57 | 6.01E-03 | cytidylate kinase-like family protein |
| J4031_09265 | 423.1 | 1.78 | 0.31 | 2.28E-07 | TlpA family protein disulfide reductase |
| J4031_07420 | 24.5 | 1.79 | 0.49 | 1.52E-03 | TonB-dependent receptor |
| J4031_01785 | 120.8 | 1.79 | 0.37 | 2.00E-05 | hypothetical protein |
| J4031_11050 | 49.5 | 1.79 | 0.65 | 1.69E-02 | RagB/SusD family nutrient uptake outer membrane protein |
| J4031_12380 | 613.2 | 1.80 | 0.48 | 1.05E-03 | TonB-dependent receptor |
| J4031_01555 | 12.2 | 1.80 | 0.60 | 9.02E-03 | smalltalk protein |
| J4031_04590 | 1199.7 | 1.80 | 0.43 | 2.05E-04 | tetratricopeptide repeat protein |
| J4031_04495 | 311.1 | 1.81 | 0.34 | 2.64E-06 | PD-(D/E)XK nuclease family protein |
| J4031_08860 | 162.8 | 1.81 | 0.64 | 1.37E-02 | TonB-dependent receptor |
| J4031_08000 | 10859.6 | 1.81 | 0.59 | 7.69E-03 | TonB-dependent receptor |
| J4031_09270 | 371.9 | 1.82 | 0.32 | 3.20E-07 | tetratricopeptide repeat protein |
| J4031_05535 | 6.2 | 1.82 | 0.67 | 1.78E-02 | smalltalk protein |
| J4031_12775 | 1945.7 | 1.83 | 0.55 | 3.63E-03 | DUF4957 domain-containing protein |
| J4031_14100 | 221.6 | 1.83 | 0.46 | 5.14E-04 | MATE family efflux transporter |
| J4031_14265 | 767.2 | 1.83 | 0.47 | 5.32E-04 | sensor histidine kinase |
| J4031_12785 | 4379.5 | 1.84 | 0.53 | 2.64E-03 | TonB-dependent receptor |
| J4031_04395 | 441.3 | 1.86 | 0.59 | 5.90E-03 | ROK family protein |
| J4031_01100 | 346.5 | 1.87 | 0.46 | 4.08E-04 | RagB/SusD family nutrient uptake outer membrane protein |
| J4031_03475 | 1113.8 | 1.87 | 0.51 | 1.35E-03 | cytidylate kinase-like family protein |
| J4031_09865 | 913.1 | 1.88 | 0.62 | 7.92E-03 | RagB/SusD family nutrient uptake outer membrane protein |
| J4031_07410 | 8.4 | 1.89 | 0.62 | 8.28E-03 | SusE domain-containing protein |
| J4031_01545 | 743.1 | 1.90 | 0.52 | 1.48E-03 | mechanosensitive ion channel family protein |
| J4031_07450 | 46.9 | 1.91 | 0.53 | 1.61E-03 | cellulase family glycosylhydrolase |
| J4031_11025 | 72.1 | 1.91 | 0.48 | 4.43E-04 | glycoside hydrolase family 3 C-terminal domain-containing protein |
| J4031_08840 | 7.1 | 1.91 | 0.70 | 1.65E-02 | DUF4861 domain-containing protein |
| J4031_07445 | 59.1 | 1.92 | 0.60 | 4.86E-03 | acetyl xylan esterase |
| J4031_07375 | 45.3 | 1.92 | 0.53 | 1.51E-03 | lipocalin family protein |
| J4031_07715 | 472.7 | 1.92 | 0.68 | 1.37E-02 | esterase |
| J4031_08855 | 112.4 | 1.93 | 0.61 | 5.80E-03 | RagB/SusD family nutrient uptake outer membrane protein |
| J4031_08065 | 663.6 | 1.94 | 0.50 | 6.95E-04 | glycoside hydrolase family 43 protein |
| J4031_06170 | 221.0 | 1.95 | 0.46 | 1.75E-04 | methylase |
| J4031_02140 | 10547.5 | 1.96 | 0.50 | 6.40E-04 | NADH peroxidase |
| J4031_09870 | 942.2 | 1.96 | 0.64 | 8.02E-03 | hypothetical protein |
| J4031_07590 | 414.8 | 1.97 | 0.58 | 2.90E-03 | prolyl oligopeptidase family serine peptidase |
| J4031_05500 | 35.8 | 1.97 | 0.39 | 5.44E-06 | hypothetical protein |
| J4031_13985 | 139.3 | 1.98 | 0.56 | 2.08E-03 | response regulator |
| J4031_11045 | 126.1 | 2.01 | 0.64 | 6.52E-03 | hypothetical protein |
| J4031_07630 | 26.6 | 2.01 | 0.74 | 1.80E-02 | RagB/SusD family nutrient uptake outer membrane protein |
| J4031_12385 | 317.4 | 2.01 | 0.50 | 4.34E-04 | RagB/SusD family nutrient uptake outer membrane protein |
| J4031_05505 | 11.6 | 2.02 | 0.53 | 7.89E-04 | hypothetical protein |
| J4031_06180 | 514.2 | 2.02 | 0.40 | 7.42E-06 | EVE domain-containing protein |
| J4031_09860 | 2544.2 | 2.03 | 0.61 | 3.51E-03 | TonB-dependent receptor |
| J4031_07510 | 157.0 | 2.03 | 0.73 | 1.49E-02 | esterase |
| J4031_04310 | 34.2 | 2.07 | 0.55 | 9.64E-04 | sulfatase-like hydrolase/transferase |
| J4031_02500 | 3.2 | 2.08 | 0.91 | 4.83E-02 | hypothetical protein |
| J4031_05530 | 210.2 | 2.08 | 0.32 | 5.37E-09 | DNA-binding protein |
| J4031_04655 | 283.8 | 2.08 | 0.55 | 9.46E-04 | RyR domain protein |
| J4031_08865 | 46.9 | 2.08 | 0.58 | 1.56E-03 | family 43 glycosylhydrolase |
| J4031_13190 | 31.3 | 2.09 | 0.43 | 1.47E-05 | hypothetical protein |
| J4031_11055 | 222.1 | 2.12 | 0.63 | 3.13E-03 | TonB-dependent receptor |
| J4031_07625 | 21.7 | 2.13 | 0.76 | 1.51E-02 | hypothetical protein |
| J4031_01565 | 115.6 | 2.14 | 0.58 | 1.18E-03 | WYL domain-containing protein |
| J4031_11040 | 59.8 | 2.14 | 0.64 | 3.47E-03 | family 16 glycosylhydrolase |
| J4031_07355 | 251.8 | 2.17 | 0.61 | 2.05E-03 | endo-1,4-beta-xylanase |
| J4031_04595 | 883.0 | 2.17 | 0.51 | 1.95E-04 | DUF4062 domain-containing protein |
| J4031_13195 | 22.3 | 2.17 | 0.51 | 2.03E-04 | antibiotic biosynthesis monooxygenase |
| J4031_14125 | 419.0 | 2.19 | 0.58 | 9.21E-04 | hypothetical protein |
| J4031_08845 | 9.1 | 2.20 | 0.69 | 5.24E-03 | heparinase II/III family protein |
| J4031_12810 | 20.5 | 2.21 | 0.80 | 1.59E-02 | TonB-dependent receptor |
| J4031_07275 | 157.4 | 2.23 | 0.62 | 1.56E-03 | family 43 glycosylhydrolase |
| J4031_04500 | 311.9 | 2.25 | 0.48 | 4.26E-05 | UvrD-helicase domain-containing protein |
| J4031_07270 | 123.5 | 2.25 | 0.70 | 5.07E-03 | family 43 glycosylhydrolase |
| J4031_01575 | 222.0 | 2.26 | 0.55 | 3.41E-04 | hypothetical protein |
| J4031_00745 | 58.2 | 2.26 | 0.64 | 1.93E-03 | DUF262 domain-containing protein |
| J4031_01320 | 1989.5 | 2.27 | 0.63 | 1.67E-03 | anaerobic C4-dicarboxylate transporter |
| J4031_13350 | 2902.2 | 2.27 | 0.55 | 2.85E-04 | response regulator |
| J4031_11030 | 28.7 | 2.29 | 0.56 | 3.09E-04 | glycoside hydrolase family 5 protein |
| J4031_04400 | 458.8 | 2.30 | 0.60 | 7.91E-04 | glycoside hydrolase family 2 |
| J4031_14130 | 472.9 | 2.30 | 0.63 | 1.36E-03 | leucine-rich repeat domain-containing protein |
| J4031_06175 | 280.2 | 2.31 | 0.47 | 1.73E-05 | hypothetical protein |
| J4031_06185 | 187.7 | 2.31 | 0.47 | 1.12E-05 | 5-methylcytosine-specific restriction endonuclease system specificity protein McrC |
| J4031_01570 | 6.8 | 2.32 | 0.70 | 3.88E-03 | hypothetical protein |
| J4031_05200 | 70.6 | 2.33 | 0.57 | 3.38E-04 | hypothetical protein |
| J4031_06145 | 777.7 | 2.34 | 0.50 | 4.02E-05 | leucine-rich repeat protein |
| J4031_07635 | 69.4 | 2.38 | 0.70 | 2.84E-03 | TonB-dependent receptor |
| J4031_07515 | 458.2 | 2.39 | 0.75 | 5.34E-03 | glycoside hydrolase family 3 C-terminal domain-containing protein |
| J4031_11790 | 4082.4 | 2.39 | 0.60 | 4.92E-04 | HAMP domain-containing histidine kinase |
| J4031_07415 | 8.6 | 2.46 | 0.66 | 1.08E-03 | RagB/SusD family nutrient uptake outer membrane protein |
| J4031_01755 | 14.6 | 2.47 | 0.49 | 7.93E-06 | hypothetical protein |
| J4031_05020 | 2880.1 | 2.48 | 0.66 | 1.01E-03 | hypothetical protein |
| J4031_00120 | 177.1 | 2.51 | 0.61 | 3.21E-04 | DUF4492 domain-containing protein |
| J4031_06325 | 726.9 | 2.51 | 0.60 | 2.47E-04 | hypothetical protein |
| J4031_07255 | 61.1 | 2.52 | 0.62 | 3.33E-04 | hypothetical protein |
| J4031_03110 | 543.3 | 2.53 | 0.63 | 3.79E-04 | hypothetical protein |
| J4031_07440 | 79.7 | 2.54 | 0.45 | 3.43E-07 | TonB-dependent receptor |
| J4031_07435 | 39.8 | 2.56 | 0.45 | 2.93E-07 | RagB/SusD family nutrient uptake outer membrane protein |
| J4031_05210 | 262.6 | 2.59 | 0.57 | 6.88E-05 | OmpA family protein |
| J4031_04990 | 73.5 | 2.59 | 0.69 | 1.07E-03 | cytochrome c biogenesis protein CcsA |
| J4031_01595 | 1737.0 | 2.62 | 0.60 | 1.31E-04 | cytidylate kinase-like family protein |
| J4031_11685 | 854.5 | 2.65 | 0.67 | 4.59E-04 | NAD-dependent dihydropyrimidine dehydrogenase subunit PreA |
| J4031_04985 | 80.8 | 2.66 | 0.69 | 6.75E-04 | cytochrome c biogenesis protein ResB |
| J4031_08225 | 1725.7 | 2.67 | 0.66 | 3.74E-04 | rubredoxin |
| J4031_05970 | 239.5 | 2.67 | 0.61 | 1.25E-04 | hypothetical protein |
| J4031_01560 | 144.0 | 2.69 | 0.46 | 2.03E-07 | DNA-binding protein |
| J4031_04855 | 1750.7 | 2.70 | 0.63 | 1.58E-04 | MBL fold metallo-hydrolase |
| J4031_12815 | 12.8 | 2.70 | 0.79 | 2.78E-03 | RagB/SusD family nutrient uptake outer membrane protein |
| J4031_07265 | 167.9 | 2.70 | 0.63 | 1.58E-04 | glycosyl hydrolase family 31 |
| J4031_07130 | 4.1 | 2.71 | 1.01 | 1.91E-02 | peptidase M15 |
| J4031_06155 | 86.9 | 2.76 | 0.52 | 2.94E-06 | hypothetical protein |
| J4031_05205 | 630.6 | 2.77 | 0.64 | 1.41E-04 | hypothetical protein |
| J4031_01055 | 386.7 | 2.80 | 0.75 | 1.12E-03 | anaerobic C4-dicarboxylate transporter |
| J4031_01550 | 451.7 | 2.83 | 0.55 | 5.37E-06 | hypothetical protein |
| J4031_00710 | 123.5 | 2.87 | 0.73 | 5.90E-04 | hypothetical protein |
| J4031_02490 | 162.4 | 2.88 | 0.71 | 3.67E-04 | hypothetical protein |
| J4031_07260 | 123.9 | 2.88 | 0.75 | 6.82E-04 | hypothetical protein |
| J4031_00750 | 19.8 | 2.91 | 0.68 | 1.58E-04 | hypothetical protein |
| J4031_04975 | 430.6 | 2.93 | 0.71 | 2.97E-04 | NapC/NirT family cytochrome c |
| J4031_05585 | 319.5 | 2.94 | 0.67 | 1.25E-04 | hypothetical protein |
| J4031_13345 | 23244.6 | 2.95 | 0.77 | 7.72E-04 | TonB-dependent receptor |
| J4031_11675 | 326.2 | 3.00 | 0.74 | 3.42E-04 | metallophosphoesterase |
| J4031_06150 | 109.8 | 3.05 | 0.57 | 2.31E-06 | hypothetical protein |
| J4031_04665 | 1013.4 | 3.07 | 0.50 | 2.59E-08 | VWA domain-containing protein |
| J4031_06160 | 160.1 | 3.07 | 0.54 | 3.10E-07 | hypothetical protein |
| J4031_00115 | 1215.7 | 3.08 | 0.92 | 3.51E-03 | cytochrome ubiquinol oxidase subunit I |
| J4031_01585 | 219.1 | 3.09 | 0.81 | 8.83E-04 | STAS domain-containing protein |
| J4031_13340 | 13049.6 | 3.12 | 0.76 | 2.87E-04 | RagB/SusD family nutrient uptake outer membrane protein |
| J4031_10620 | 1350.3 | 3.16 | 0.76 | 2.68E-04 | alanine dehydrogenase |
| J4031_11680 | 231.9 | 3.16 | 0.73 | 1.46E-04 | hypothetical protein |
| J4031_00715 | 222.2 | 3.21 | 0.81 | 5.28E-04 | hypothetical protein |
| J4031_07520 | 56764.2 | 3.24 | 0.77 | 2.35E-04 | hypothetical protein |
| J4031_04695 | 118.2 | 3.32 | 1.01 | 4.10E-03 | hypothetical protein |
| J4031_05580 | 440.7 | 3.34 | 0.71 | 3.24E-05 | hypothetical protein |
| J4031_04690 | 146.1 | 3.36 | 0.98 | 2.67E-03 | two pore domain potassium channel family protein |
| J4031_00725 | 271.2 | 3.36 | 0.93 | 1.61E-03 | PD-(D/E)XK nuclease family protein |
| J4031_04980 | 1267.4 | 3.36 | 0.72 | 3.50E-05 | ammonia-forming cytochrome c nitrite reductase |
| J4031_09615 | 153.1 | 3.38 | 0.82 | 2.96E-04 | RagB/SusD family nutrient uptake outer membrane protein |
| J4031_07460 | 1264.3 | 3.45 | 1.17 | 1.02E-02 | TonB-dependent receptor |
| J4031_08825 | 1296.6 | 3.46 | 0.97 | 1.66E-03 | TonB-dependent receptor |
| J4031_00110 | 812.2 | 3.49 | 0.98 | 1.81E-03 | cytochrome d ubiquinol oxidase subunit II |
| J4031_09610 | 133.3 | 3.50 | 0.75 | 4.36E-05 | DUF4960 domain-containing protein |
| J4031_02495 | 224.5 | 3.51 | 0.72 | 1.73E-05 | hypothetical protein |
| J4031_09605 | 70.1 | 3.51 | 0.81 | 1.34E-04 | glycoside hydrolase family 32 protein |
| J4031_07465 | 1014.6 | 3.58 | 1.15 | 6.70E-03 | RagB/SusD family nutrient uptake outer membrane protein |
| J4031_05350 | 1087.4 | 3.58 | 0.72 | 1.15E-05 | (Fe-S)-binding protein |
| J4031_04680 | 878.1 | 3.67 | 1.04 | 2.11E-03 | SUMF1/EgtB/PvdO family nonheme iron enzyme |
| J4031_09620 | 458.6 | 3.67 | 0.77 | 2.75E-05 | TonB-dependent receptor |
| J4031_12755 | 3470.3 | 3.68 | 0.71 | 3.94E-06 | L-lactate permease |
| J4031_00720 | 89.2 | 3.68 | 1.01 | 1.42E-03 | hypothetical protein |
| J4031_01045 | 791.9 | 3.69 | 0.96 | 7.79E-04 | aspartate ammonia-lyase |
| J4031_08830 | 1217.3 | 3.74 | 0.96 | 6.55E-04 | RagB/SusD family nutrient uptake outer membrane protein |
| J4031_04675 | 830.6 | 3.74 | 1.02 | 1.34E-03 | hypothetical protein |
| J4031_04685 | 220.0 | 3.76 | 1.11 | 3.08E-03 | hypothetical protein |
| J4031_04670 | 2198.5 | 3.77 | 0.94 | 4.22E-04 | hypothetical protein |
| J4031_00730 | 160.4 | 3.78 | 0.96 | 5.04E-04 | phospholipase |
| J4031_05575 | 283.7 | 3.79 | 0.60 | 1.43E-08 | hypothetical protein |
| J4031_05355 | 1272.3 | 3.84 | 0.79 | 1.73E-05 | lactate utilisation protein |
| J4031_09595 | 2215.7 | 3.93 | 0.97 | 3.74E-04 | DUF4980 domain-containing protein |
| J4031_09585 | 1155.4 | 4.08 | 0.99 | 3.00E-04 | carbohydrate kinase |
| J4031_01050 | 86.0 | 4.11 | 1.16 | 2.02E-03 | hypothetical protein |
| J4031_05360 | 665.0 | 4.11 | 0.80 | 4.81E-06 | LUD domain-containing protein |
| J4031_09635 | 1046.6 | 4.12 | 1.05 | 5.37E-04 | DUF4960 domain-containing protein |
| J4031_09590 | 1233.0 | 4.25 | 1.02 | 2.57E-04 | MFS transporter |
| J4031_09630 | 2552.4 | 4.38 | 1.02 | 1.68E-04 | DUF4975 domain-containing protein |
| J4031_09645 | 9664.1 | 4.48 | 1.02 | 1.15E-04 | TonB-dependent receptor |
| J4031_09640 | 2467.3 | 4.54 | 1.03 | 1.03E-04 | RagB/SusD family nutrient uptake outer membrane protein |

^1^ Propionate pathway transcripts are highlighted in bold. ^2^ Calculated in DESeq2 as the mean normalised counts across all samples. ^3^ Q-values were calculated using the Benjamini-Hochberg procedure.

**Table S7. Differentially abundant KHP1 proteins between cobalamin treatments**

| Protein ID^1^ | Log_2_fold change (+B_12_/-B_12_) | t-test statistic (+B_12_/-B_12_) | Student's t-test q-value^2^ | Gene annotation |
| --- | --- | --- | --- | --- |
| QVJ81054.1 | -6.72 | -10.93 | 8.00E-04 | sirohydrochlorin cobaltochelatase |
| QVJ81053.1 | -5.26 | -20.92 | 0.00E+00 | TonB-dependent receptor |
| QVJ82096.1 | -3.31 | -7.44 | 3.14E-03 | O-acetylhomoserine aminocarboxypropyltransferase/cysteine synthase |
| QVJ81056.1 | -3.27 | -13.17 | 1.00E-03 | precorrin-3B C methyltransferase |
| QVJ79760.1 | -2.83 | -14.03 | 1.33E-03 | DUF4465 containing protein |
| QVJ81055.1 | -2.20 | -18.03 | 0.00E+00 | TonB-dependent receptor |
| QVJ81057.1 | -2.20 | -10.28 | 6.67E-04 | cobyric acid synthase |
| QVJ82080.1 | -1.20 | -7.08 | 4.27E-03 | response regulator transcription factor |
| QVJ81973.1 | -1.20 | -3.98 | 4.05E-02 | 5'/3'-nucleotidase SurE |
| QVJ82095.1 | -1.05 | -7.06 | 4.25E-03 | cysteine synthase A |
| QVJ80504.1 | **1.65** | **9.66** | **1.00E-03** | **methylmalonyl-CoA mutase** |
| QVJ80503.1 | **1.71** | **9.54** | **8.89E-04** | **methylmalonyl-CoA mutase small subunit** |
| QVJ80162.1 | 1.75 | 5.91 | 8.17E-03 | glycine cleavage system protein GcvH |

^1^ Propionate pathway proteins are highlighted in bold. ^2^ Q-values were calculated using the permutation-based FDR method (250 randomisations).

**Table S8. Homologues of methylmalonyl-CoA mutase accessory proteins**

| **Uniprot ID** | **protein** | **Organism** | **KHP1 match (protein ID – aa identity)^1^** | **KHP1 annotation** |
| --- | --- | --- | --- | --- |
| C5AP93 | methylmalonyl-CoA mutase associated GTPase | *Methylobacterium extorquens* AM1 | QVJ81811 – 48.8% | methylmalonyl-CoA mutase-associated GTPase MeaB |
| Q8ZNR5 | corrinoid adenosyltransferase pduO | *Salmonella typhimurium* | QVJ80035 – 40.6% | cob(I)yrinic acid a, c-diamide adenosyltransferase |
| P65643 | corrinoid adenosyltransferase EutT | *E. coli* K12 | no matches |  |
| Q9I472 | corrinoid adenosyltransferase cobO | *Pseudomonas aeruginosa* | no matches |  |
| Q1LJ80 | corrinoid adenosyltransferase cobO (pduO-type) | *Cupriavidus metallidurans* | QVJ80035– 41.3% | cob(I)yrinic acid a, c-diamide adenosyltransferase |
| P0A9H5 | corrinoid adenosyltransferase btuR | *E. coli* K12 | no matches |  |
| P29930 | corrinoid adenosyltransferase | *Sinorhizobium* sp. | no matches |  |
| P31570 | corrinoid adenosyltransferase cobA | *Salmonella typhimurium* | no matches |  |
| B1VB74 | corrinoid adenosyltransferase pduO | *Citrobacter Freundii* | QVJ80035– 39.8% | cob(I)yrinic acid a, c-diamide adenosyltransferase |
| O34899 | corrinoid adenosyltransferase yvqK | *Bacillus subtilis* | QVJ80035– 39.3% | cob(I)yrinic acid a, c-diamide adenosyltransferase |
| P45515 | corrinoid adenosyltransferase | *Citrobacter Freundii* | QVJ80035– 38.2% | cob(I)yrinic acid a, c-diamide adenosyltransferase |
| P9WP98 | corrinoid adenosyltransferase | *Mycobacterium tuberculosis* | QVJ80035– 34.9% | cob(I)yrinic acid a, c-diamide adenosyltransferase |
| P53523 | corrinoid adenosyltransferase | *Mycobacterium leprae* TN | QVJ80035– 35.9% | cob(I)yrinic acid a, c-diamide adenosyltransferase |
| P64804 | corrinoid adenosyltransferase | *Mycobacterium bovis* | QVJ80035– 34.9% | cob(I)yrinic acid a, c-diamide adenosyltransferase |
| P9WP99 | corrinoid adenosyltransferase | *Mycobacterium tuberculosis* | QVJ80035– 34.9% | cob(I)yrinic acid a, c-diamide adenosyltransferase |
| Q9I472 | corrinoid adenosyltransferase (cobO) | *Pseudomonas aeruginosa* | no matches |  |
| P0A9H6 | corrinoid adenosyltransferase (BtuR) | *E. coli* 06:H1 | no matches |  |
| P65644 | corrinoid adenosyltransferase EutT | *E. coli* 06:H1 | no matches |  |
| Q9ZFV4 | corrinoid adenosyltransferase EutT | *Salmonella typhimurium* LT2 | no matches |  |

^1^ Determined by BLASTP (default settings).


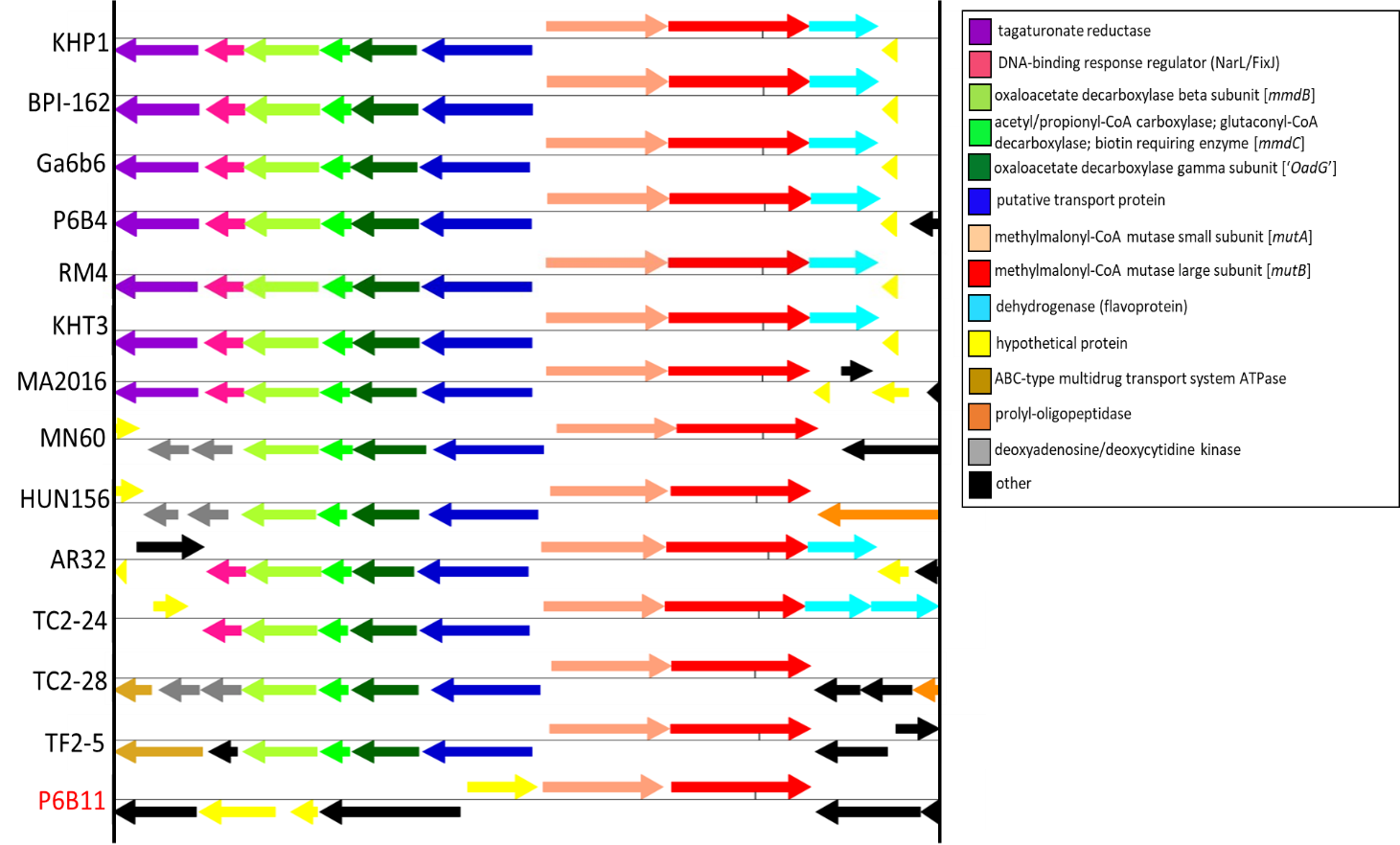


**Fig. S1.** **Conserved arrangement of methylmalonyl-CoA mutase and methylmalonyl-CoA decarboxylase genes across propionate-producing *‘Prevotella* 1’ strains.** Gene maps of regions of each screened contig containing methylmalonyl-CoA mutase genes, coloured by annotation. Gene names classified as ‘other’ represent annotations present on only one extracted fragment. Black markers underneath methylmalonyl-CoA mutase large subunit genes represent 10 kb markers from the left side of each panel.


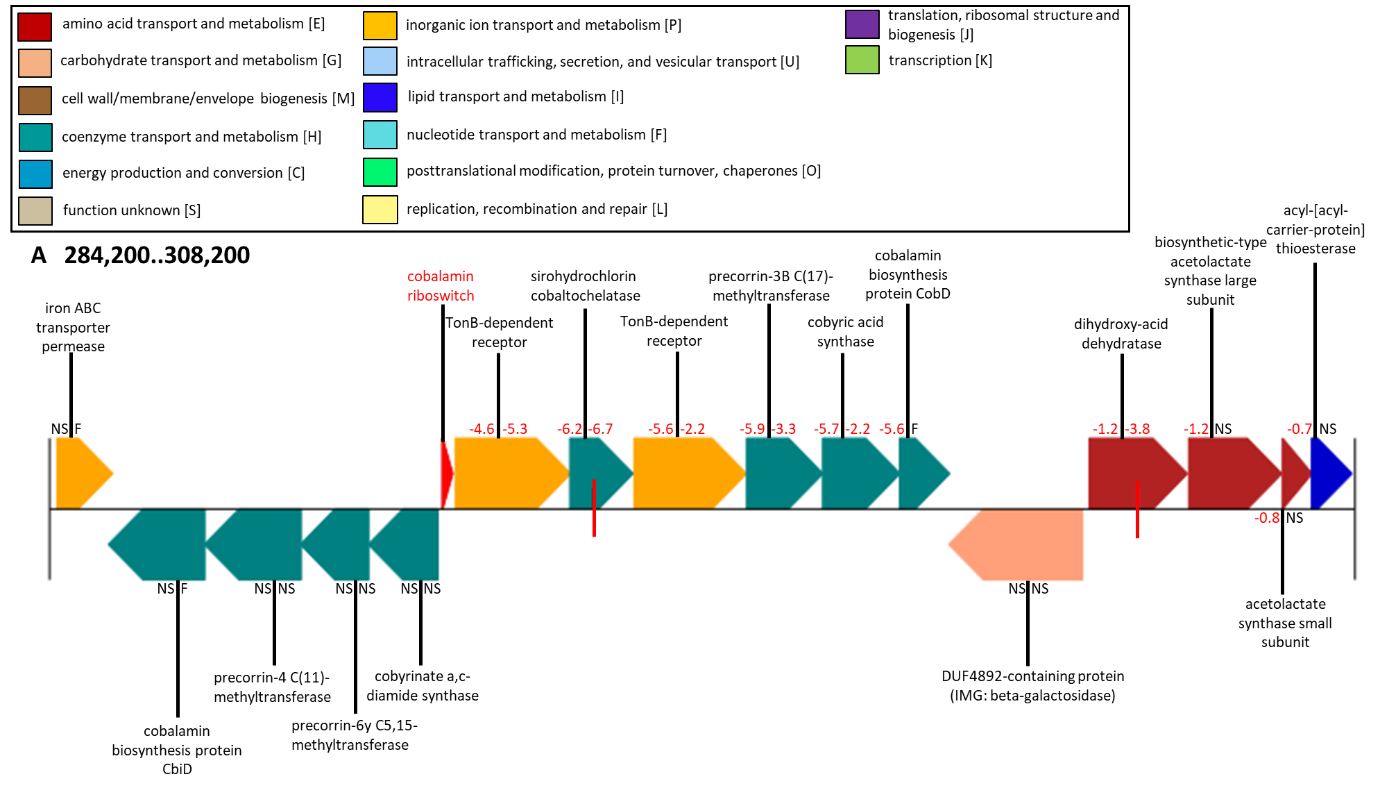


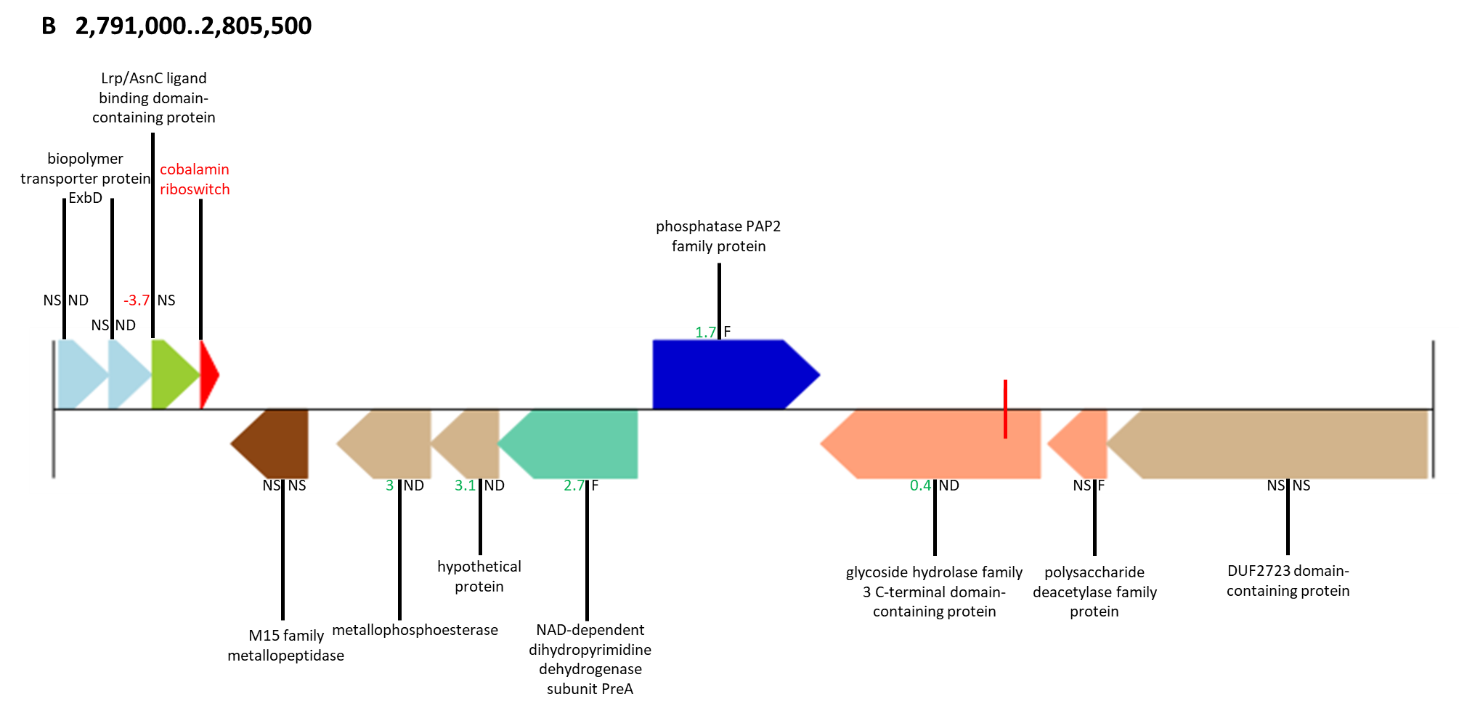


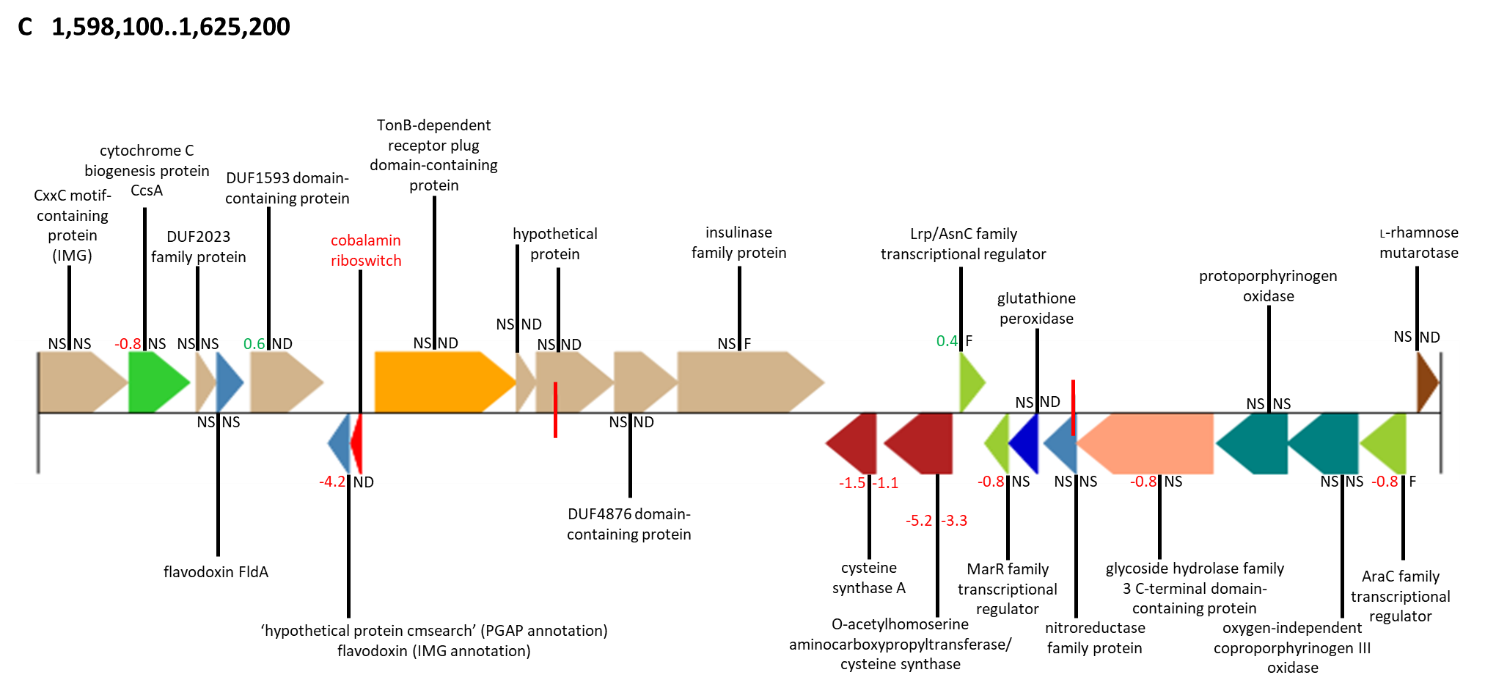


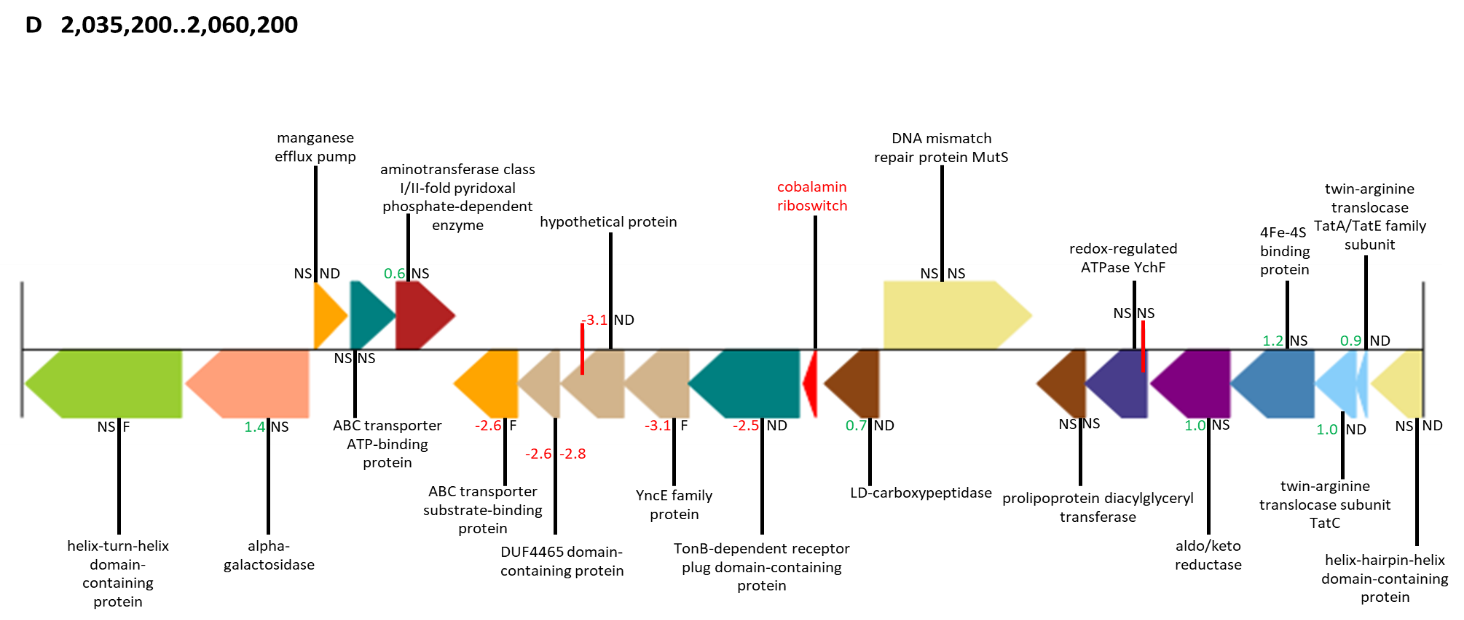


**Fig. S2. Gene and protein expression of genes in close proximity to putative cobalamin family riboswitches.** In each panel genome coordinates of each fragment of the KHP1 genome are shown. Numbers at the markers for each gene represent log_2_ fold changes of transcript abundances (left) and protein abundances (right) between each treatment. Positive values represent increased abundance in the cobalamin-supplemented compared to non-supplemented media. NS = not significant (DESeq2 (transcriptome)/two sample t-test (proteome); FDR-adjusted *P* > 0.05); ND = protein not detected in proteome data; F = protein filtered out during quality control. Smaller red vertical markers represent 10 kb increments from the left of each fragment.


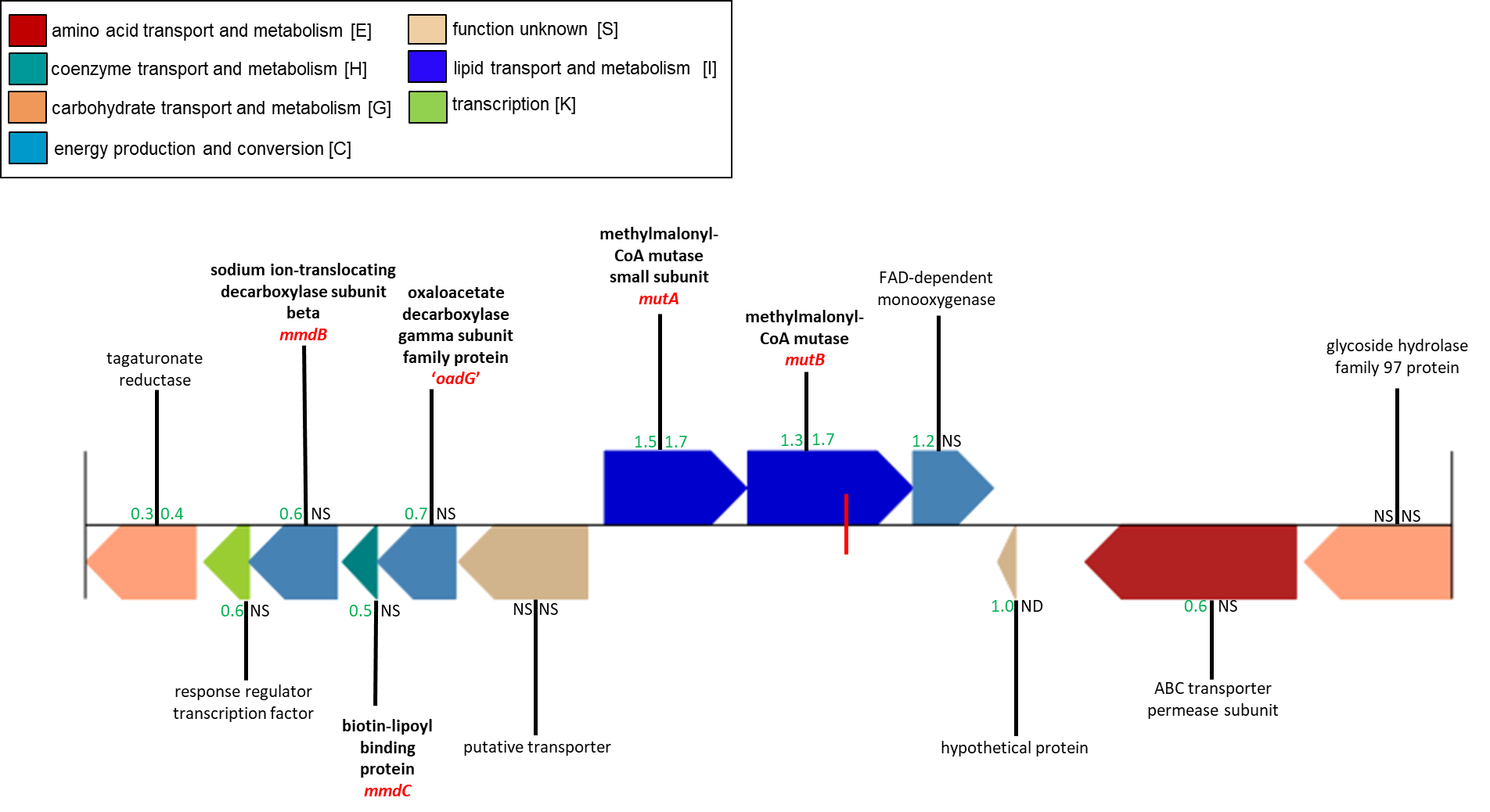


**Fig. S3. Impact of cobalamin on the expression of genes encoding the upregulated methylmalonyl-CoA decarboxylase/methylmalonyl-CoA mutase subunits and nearby genes.** Numbers at the markers for each gene represent log_2_ fold changes of transcript abundances (left) and protein abundances (right) between each treatment. Positive values represent increased abundance in the cobalamin-supplemented compared to non-supplemented media. NS = not significant (DESeq2 (transcriptome)/two sample t-test (proteome); FDR-adjusted *P* > 0.05); ND = protein not detected in proteome data. Smaller red vertical markers represent 10 kb increments from the left of the fragment
